# Implication of genetic variants in primary microRNA processing sites in the risk of multiple sclerosis

**DOI:** 10.1016/j.ebiom.2022.104052

**Published:** 2022-05-10

**Authors:** Michael Hecker, Brit Fitzner, Elena Putscher, Margit Schwartz, Alexander Winkelmann, Stefanie Meister, Ales Dudesek, Dirk Koczan, Peter Lorenz, Nina Boxberger, Uwe Klaus Zettl

**Affiliations:** a Department of Neurology, Division of Neuroimmunology, Rostock University Medical Center, Gehlsheimer Str. 20, Rostock 18147, Germany; b Institute of Immunology, Rostock University Medical Center, Schillingallee 70, Rostock 18057, Germany

**Keywords:** Multiple sclerosis, Genetic risk, Single-nucleotide polymorphisms, MicroRNAs, B cells, Expression quantitative trait loci, Primary microRNA processing, MicroRNA target genes, Immune reconstitution therapy, ANOVA, analysis of variance, APC, allophycocyanin, bp, base pairs, BV, brilliant violet, CNS, central nervous system, *C*_T_, threshold cycle, EAE, experimental autoimmune encephalomyelitis, EBV, Epstein-Barr virus, EDSS, Expanded Disability Status Scale, EDTA, ethylenediaminetetraacetic acid, eQTL, expression quantitative trait locus, EUR, European, FITC, fluorescein isothiocyanate, GEO, Gene Expression Omnibus, GLuc, *Gaussia* luciferase, GWAS, genome-wide association study, h, hours, HGNC, HUGO Gene Nomenclature Committee, HLA, human leukocyte antigen, IRT, immune reconstitution therapy, isomiR, microRNA isoform, kb, kilobase, LD, linkage disequilibrium, MAF, minor allele frequency, mfe, minimum free energy, miR/MIR/miRNA, microRNA, mRNA, messenger RNA, MS, multiple sclerosis, mv, missing values, n/N, number, ng, nanogram, nt, nucleotide, OR, odds ratio, PBMC, peripheral blood mononuclear cells, PE, phycoerythrin, PerCP, peridinin-chlorophyll, PPMS, primary progressive multiple sclerosis, pri-miRNA, primary microRNA, qPCR, quantitative polymerase chain reaction, RA, risk allele, RPM, reads per million, RRMS, relapsing-remitting multiple sclerosis, RT, reverse transcription, SD, standard deviation, SEAP, secreted alkaline phosphatase, SNP, single-nucleotide polymorphism, SPMS, secondary progressive multiple sclerosis, sQTL, splicing quantitative trait locus, TAC, Transcriptome Analysis Console, TC, transcript cluster, T_h_17, T helper 17 cell, UNG, uracil-N-glycosylase, UTR, untranslated region

## Abstract

**Background:**

Multiple sclerosis (MS) is a chronic inflammatory disease of the central nervous system with a well-established genetic contribution to susceptibility. Over 200 genetic regions have been linked to the inherited risk of developing MS, but the disease-causing variants and their functional effects at the molecular level are still largely unresolved. We hypothesised that MS-associated single-nucleotide polymorphisms (SNPs) affect the recognition and enzymatic cleavage of primary microRNAs (pri-miRNAs).

**Methods:**

Our study focused on 11 pri-miRNAs (9 primate-specific) that are encoded in genetic risk loci for MS. The levels of mature miRNAs and potential isoforms (isomiRs) produced from those pri-miRNAs were measured in B cells obtained from the peripheral blood of 63 MS patients and 28 healthy controls. We tested for associations between SNP genotypes and miRNA expression in *cis* using quantitative trait locus (*cis*-miR-eQTL) analyses. Genetic effects on miRNA stem-loop processing efficiency were verified using luciferase reporter assays. Potential direct miRNA target genes were identified by transcriptome profiling and computational binding site assessment.

**Findings:**

Mature miRNAs and isomiRs from hsa-mir-26a-2, hsa-mir-199a-1, hsa-mir-4304, hsa-mir-4423, hsa-mir-4464 and hsa-mir-4492 could be detected in all B-cell samples. When MS patient subgroups were compared with healthy controls, a significant differential expression was observed for miRNAs from the 5’ and 3’ strands of hsa-mir-26a-2 and hsa-mir-199a-1. The *cis*-miR-eQTL analyses and reporter assays pointed to a slightly more efficient Drosha-mediated processing of hsa-mir-199a-1 when the MS risk allele T of SNP rs1005039 is present. On the other hand, the MS risk allele A of SNP rs817478, which substitutes the first C in a CNNC sequence motif, was found to cause a markedly lower efficiency in the processing of hsa-mir-4423. Overexpression of hsa-mir-199a-1 inhibited the expression of 60 protein-coding genes, including *IRAK2, MIF, TNFRSF12A* and *TRAF1*. The only target gene identified for hsa-mir-4423 was *TMEM47*.

**Interpretation:**

We found that MS-associated SNPs in sequence determinants of pri-miRNA processing can affect the expression of mature miRNAs. Our findings complement the existing literature on the dysregulation of miRNAs in MS. Further studies on the maturation and function of miRNAs in different cell types and tissues may help to gain a more detailed functional understanding of the genetic basis of MS.

**Funding:**

This study was funded by the Rostock University Medical Center (FORUN program, grant: 889002), Sanofi Genzyme (grant: GZ-2016-11560) and Merck Serono GmbH (Darmstadt, Germany, an affiliate of Merck KGaA, CrossRef Funder ID: 10.13039/100009945, grant: 4501860307). NB was supported by the Stiftung der Deutschen Wirtschaft (sdw) and the FAZIT foundation. EP was supported by the Landesgraduiertenförderung Mecklenburg-Vorpommern.

**Research in context**

**Evidence before this study**

Genome-wide association studies of multiple sclerosis (MS) have identified over 200 risk loci across the human genome. However, our understanding of disease architecture is still far from complete. Discerning the causal variants and gaining functional insights are essential steps towards bridging the gap between genotypes and clinical phenotypes. Some disease susceptibility loci encode microRNAs (miRNAs). We suspected that the genotype of MS-associated single-nucleotide polymorphisms (SNPs) might influence the enzymatic processing and thus the expression of such miRNAs and their target genes.

**Added value of this study**

We used B cells from the peripheral blood of MS patients and healthy controls to measure selected mature miRNAs and isoforms produced from primary miRNA transcripts that originate from genetic risk loci for MS. We found that miRNAs from both the 5′ and the 3′ strand of hsa-mir-26a-2 and hsa-mir-199a-1 were differentially expressed between the groups. A higher expression of miR-199a miRNAs was observed in the presence of the MS risk allele of SNP rs1005039, whereas the risk allele of SNP rs817478 was associated with a lower expression of hsa-miR-4423-5p. These genetic effects on primary miRNA processing were verified using reporter assays. Moreover, we identified known and new potential direct target genes of these miRNAs.

**Implications of all evidence available**

We show that MS-associated SNPs in primary miRNA processing sites can affect the expression of mature miRNAs. These findings contribute to a better understanding of the molecular mechanisms underlying the pathogenesis of MS. Further cell type-specific studies on transcriptional regulation and RNA processing are necessary to elucidate the functional nature of the numerous genetic determinants of disease susceptibility. These efforts will help to define the specific cell types and complex regulatory networks causally involved in MS.

Alt-text: Unlabelled box

## Introduction

Multiple sclerosis (MS) is a chronic, inflammatory and neurodegenerative disease of the central nervous system (CNS).^1^ The pathological hallmark of MS is the formation of demyelinating lesions in the brain and spinal cord, which are caused by the infiltration of immune cells (e.g., T cells and B cells).^2^ Clonal expansion of B cells in MS CNS usually results in intrathecal antibody production.^3^ The clinical presentation and course of MS are heterogeneous.^4^ Symptoms of MS include mobility restrictions (oftentimes due to paresis and spasticity), visual impairment, bladder and bowel problems, pain, depression, cognitive deficits and fatigue.^5^ Different courses of MS are distinguished^6^: relapsing-remitting MS (RRMS), secondary progressive MS (SPMS) and primary progressive MS (PPMS). In the early phase of the disease, 85–90% of the patients have RRMS with reversible episodes of neurological deficits (known as relapses) lasting several days or weeks. Over time, RRMS patients tend to stop having relapses but continue to develop permanent neurological deficits, which is referred to as the SPMS stage.^7^ A minority of patients (10–15%) have PPMS, with progression of disability without acute relapses from disease onset. RRMS is typically diagnosed in young adults between 20 years and 35 years of age, whereas PPMS usually begins at ∼40 years of age.^1^^,^^8^ Moreover, it has been shown that the female-to-male ratio is 2.5:1 for patients with RRMS/SPMS and 1.2:1 for patients with PPMS.^9^

There is still no curative therapy for MS, but a better understanding of the immunological and neurobiological processes underlying MS has led to the development of treatments that can significantly reduce disease activity.^1^ For instance, monthly infusions of the monoclonal antibody natalizumab, which inhibits the transendothelial migration of lymphocytes into the CNS by blocking their attachment to cell adhesion proteins, proved highly effective in preventing relapses and slowing progression of disability.^10^^,^^11^ The newer so-called immune reconstitution therapies (IRTs) even have the potential to induce long-term drug-free remission in many patients.^12^ With IRTs, components of the adaptive immune system are depleted to allow for renewal. Typical representatives of IRTs are alemtuzumab and cladribine, which are administered in annual treatment courses.^13^ A common mechanism of action is the preferential depletion of memory B cells.^14^ Following depletion, the circulating CD19^+^ B cells repopulate within 6-9 months [14-16]. B cells are key players as they mediate cytokine production, antigen presentation, antibody synthesis and the formation of ectopic follicles at sites of inflammation.^3^ However, the reader is referred to the literature for a more comprehensive overview on the broad spectrum of disease-modifying treatments available for MS^17^, ^18^, ^19^ and on the roles of B cells in MS.^20^^,^^21^

The exact causes of MS are still unknown, but it is well established that the individual risk of developing MS is determined by interactions of genetic and non-genetic factors. Environmental and lifestyle factors that have been found to be associated with MS risk include Epstein-Barr virus (EBV) infection, cigarette smoking, adolescent obesity and vitamin D deficiency.^22^^,^^23^ The strongest genetic susceptibility variants for MS are located within the human leukocyte antigen (HLA) region. In particular, the HLA-DRB1*15:01 allele confers an increased risk of MS with an odds ratio (OR) of 3.92, whereas the HLA-A*02:01 allele has protective effects (OR=0.67).^24^ In addition, more than 200 non-HLA MS risk loci with ORs ranging from 1.06 to 2.06 have been identified in large-scale genetic studies with more than 100,000 subjects of primarily European ancestry.^25^^,^^26^ Cell type-specific enrichments of MS genetic signals have pointed to prominent roles of microglia^26^ as well as T_h_17 cells and memory B cells^27^ in the pathogenesis of MS. However, the disease-causative variants remain largely unclear, as different single-nucleotide polymorphisms (SNPs) in linkage disequilibrium (LD) can explain the association with MS.^28^ Most disease-associated SNPs are expected to capture regulatory effects, which means that they are colocalised with quantitative trait loci (QTLs), e.g., for gene expression (eQTLs) and splicing (sQTLs).^29^ In contrast to extensive investigations of mRNA eQTLs and sQTLs across many human tissues and cell types,^30^, ^31^, ^32^ there are still relatively few studies on microRNA (miRNA) expression QTLs (miR-eQTLs).^33^^,^^34^

MicroRNAs are small non-coding RNAs about 22 nt in length that act as post-transcriptional regulators of gene expression.^35^ A total of 2,883 human mature miRNAs are currently listed in the miRBase database release 22.^36^ Approximately half of all known miRNAs originate from intragenic regions.^37^ However, the expression of miRNA and host gene can be uncoupled.^38^ Mature miRNAs are processed from stem-loop regions of longer primary miRNA (pri-miRNA) transcripts in distinct maturation steps^39^: A key step is the cleavage of the miRNA stem-loop by Microprocessor,^40^^,^^41^ a nuclear complex of the RNase Drosha and its essential cofactor DGCR8. This leads to a small hairpin-shaped precursor miRNA, which is exported into the cytoplasm where it is cleaved by the RNase Dicer. One strand of the resulting miRNA duplex is loaded into the so-called RNA-induced silencing complex, which mediates the decay and/or translational repression of target transcripts that contain a binding site for the mature miRNA. Alternative cleavage sites and post-maturation sequence modifications can give rise to isoforms of miRNAs (isomiRs) that differ in sequence from the canonical mature miRNA.^42^

Sequence motifs in the miRNA stem-loop region are known to influence the processing by Microprocessor. These are a UG motif at position ∼−14/−13 from the 5′ Drosha cleavage site, a GUG/UGU motif in the apical loop and a ∼16–21 nt downstream CNNC motif,^43^ which is bound by the proteins DDX17 and SRSF3.^44^^,^^45^ Roughly 80% of all conserved human miRNA sites contain at least one of these motifs.^44^ Further determinants of an efficient miRNA processing are a GNNU motif and a mismatched GHG motif in the basal stem region and an extensive base pairing of the ∼35-bp stem.^41^^,^^46^ However, yet unknown sequence motifs and accessory proteins are thought to contribute to the Drosha-mediated cleavage.^47^ SNPs that are located in features required for the recognition of pri-miRNAs can affect their processing. First studies could show a dependence of the expression of miRNAs on the genotype of MS-associated SNPs in *cis*: For carriers of the risk alleles of the SNPs rs2910164 (C) and rs1414273 (C), an increased expression of hsa-miR-146a-5p and hsa-miR-548ac-3p, respectively, was described in blood cells.^48^, ^49^, ^50^

In this study, we conducted a more systematic investigation of miRNAs derived from genetic loci associated with MS risk. To this end, we measured the levels of prioritised mature miRNAs and isomiRs in B cells from MS patients (n=63) and healthy controls (n=28) and analysed whether their expression is subject to *cis*-miR-eQTL effects. We then used luciferase reporter assays to verify whether the enzymatic processing of selected miRNA stem-loops is indeed causally related to the genotype of the proximal SNPs. Moreover, we aimed to identify new target genes that are post-transcriptionally regulated by the miRNAs under scrutiny. Our study provides insights into the molecular mechanisms underlying the pathogenesis of MS by uncovering the putative functional role of genetic variants implicated in disease susceptibility.

## Methods

### Selection of microRNAs from MS-associated genetic regions

**Figure 1:**
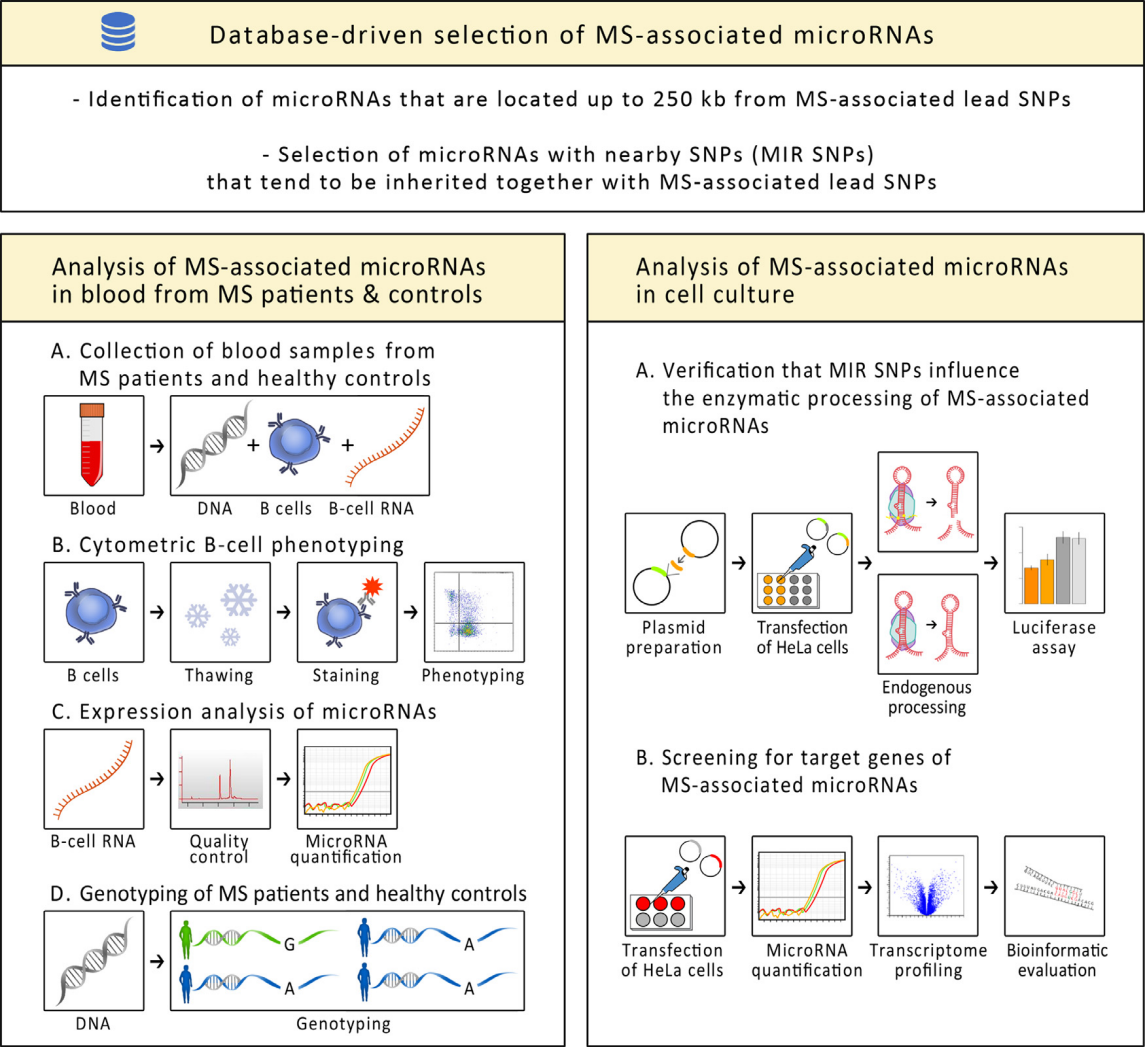
Overview of the study. This study comprised three parts: First, primary miRNAs encoded in genetic risk loci for MS were identified by a database-driven approach. Second, DNA, B cells and B-cell RNA were collected from MS patients as well as from healthy controls. Flow cytometry was used to phenotype B-cell subpopulations. The expression levels of mature miRNAs and isomiRs were measured using specific qPCR assays. The subjects were genotyped with respect to MS-associated SNPs within the miRNA-coding regions. Third, the effect of the SNPs on the enzymatic cleavage of the primary miRNAs by the Microprocessor complex was investigated using luciferase reporter assays. Transcriptome profiling was used to identify miRNA target genes. MIR=microRNA, MS=multiple sclerosis, qPCR=quantitative polymerase chain reaction, SNP=single-nucleotide polymorphism.

A database-driven approach was employed to prioritise MS-associated genetic variants in potential pri-miRNA processing sites (Figure 1). First, we identified all human miRNA stem-loops that are recorded in the miRBase database (release 21)^36^ and that are encoded within a distance of <250 kb to the 233 MS-associated lead SNPs, which have been reported in the most recent genome-wide association study (GWAS) in MS.^26^ Second, we used the LDproxy and LDpair modules of the web-based tool LDlink (release 3.0)^51^ to check whether the miRNA loci contain SNPs (hereinafter referred to as MIR SNPs) that are in LD with the MS lead SNPs. More specifically, we excluded miRNA-encoding regions for which all proximal SNPs had an *r^2^*<0.1 in the European subpopulation of the 1000 Genomes panel,^52^ and we selected genetic variants with *D'*>0.7 that are located within a miRBase-annotated stem-loop sequence or 50 bp upstream or downstream of the putative Drosha cleavage sites and that had a minor allele frequency (MAF) >1% according to dbSNP version 150.^53^ If the MIR SNPs were not included in the LDlink tool, adjacent SNPs were considered as proxy. Finally, we excluded SNPs that presumably do not affect the canonical pri-miRNA processing by Drosha (e.g., SNPs in the vicinity of mirtrons^39^).

The prioritised pri-miRNAs may give rise to different miRNA isoforms that differ at the 5′ or 3′ end from the miRBase-annotated mature miRNA forms. Mature miRNAs may also originate from both arms of a precursor miRNA, even if not explicitly recorded in the miRBase database.^36^ Therefore, we extracted potential isomiRs from the IsomiR Bank database^54^ and ordered them by the cumulative reads per million (RPM) values. Additionally, we inspected the normalised miRNA expression data pileup plots provided in the miRCarta database version 1.1.^55^ Based on this information, we derived up to 2 potential isomiRs with a length of >16 bases for each arm of each precursor miRNA. The isomiRs were named following the nomenclature by Cloonan et al.^56^

The web-based tools RNAfold^57^ and forna^58^ were used to visualise the predicted minimum free energy (mfe) secondary structures of selected miRNA stem-loop sequences.

### Study groups and samples

We were interested in examining whether the selected miRNAs are dysregulated in B cells of patients with MS and whether their levels are related to the genotype of MS-associated SNPs. For this purpose, 20 ml of peripheral blood were taken from MS patients during regular clinical visits as well as from healthy donors at the Rostock University Medical Center between July 2015 and December 2019. Individuals younger than 18 years of age were excluded from this observational study. The diagnosis of MS has been confirmed according to the revised McDonald criteria.^59^ Neurological impairment was rated using the Expanded Disability Status Scale (EDSS).^60^ Routine medical care was provided to all patients. Hence, the patients were treated and monitored according to the guidelines and recommendations of the German Society of Neurology.

The blood samples were collected from a peripheral vein into tubes with ethylenediaminetetraacetic acid (EDTA) as anticoagulant. Immediately following the blood withdrawal, each sample was processed to obtain DNA and B cells (Figure 1). DNA was purified from 200 µl of whole blood using the QIAamp DNA Blood Mini Kit (Qiagen) and stored at -20^°^C. Peripheral blood mononuclear cells (PBMC) were isolated using Ficoll density gradient separation (Histopaque-1077, Sigma-Aldrich). Untouched B cells were then enriched from the PBMC by negative selection using the Pan B Cell Isolation Kit (Miltenyi Biotec). Accordingly, non-B cells were magnetically labelled with a cocktail of biotin-conjugated monoclonal antibodies and anti-biotin MicroBeads and then removed by magnetic separation following manufacturer's instructions. The number of isolated B cells was counted under a microscope using Neubauer chambers. From each sample, two aliquots of 100,000 B cells were cryopreserved in freezing medium (Biofreeze, Biochrom) in the vapor phase of liquid nitrogen at below -140^°^C. From the remaining B cells, total RNA was preserved using the miRNeasy Mini Kit (Qiagen) in combination with the RNase-free DNase set (Qiagen). The RNA was stored at -80^°^C.

### Flow cytometry

The purities of freshly isolated B cells were assessed by flow cytometry using BD FACSCalibur and BD FACSAria instruments and CD19 or CD20 antibodies conjugated to fluorescein isothiocyanate (FITC) or phycoerythrin (PE) (RRIDs: AB_2726271, AB_2726142 and AB_2656064) (Miltenyi Biotec). The acquired data were analysed using Flowing Software version 2.5.1 (Turku Bioscience).

To characterise the samples in more depth, we aimed to acquire information on the composition of the peripheral B cells from the healthy subjects and the patients with MS. The B-cell phenotyping was done with the cryopreserved B cells using a BD FACSAria Illu system. For this purpose, the B cells were quickly thawed in a 37 ^°^C water bath, washed with phosphate buffered saline and then stained with the fluorochrome-conjugated antibodies CD19-PerCP (RRID: AB_893276), CD23-BV421 (RRID: AB_2562460), CD27-PE (RRID: AB_314299), CD38-PE/Dazzle594 (RRID: AB_2564104) (all from BioLegend), CD21-APC (RRID: AB_2727123), CD24-APC-Vio770 (RRID: AB_2656566) (Miltenyi Biotec) and IgD-BV750 (RRID: AB_2868411) (BD). Zombie Green dye was used for live/dead staining of the cells (BioLegend). FcR blocking reagent (Miltenyi Biotec) was used to block unspecific binding of antibodies to Fc receptors. In each staining batch, 2 million cells of pooled PBMC from healthy subjects were used as an internal control. Data acquisition and compensation calculations were performed using the BD FACSDiva software version 8.0.2.

The data were further processed in the FlowJo software version 10 from BD. The FlowJo plugin CytoNorm version 0.9^61^ was applied for normalising batch effects based on the data of the internal controls. The plugin FlowAI version 2.1^62^ was applied for checking the data quality (e.g., the steadiness of the flow rate) and for detecting and removing outlier events. The gating strategy for the identification of B cell subsets was adapted from Morbach et al*.*^63^ First, live single CD19^+^ cells were determined based on their forward and side scatter properties and the signals for the live/dead and CD19 markers. We then gated on CD27^−^IgD^+^ naive B cells, CD27^+^IgD^+^ non-switched memory B cells, CD27^+^IgD^−^ switched memory B cells, CD27^−^IgD^−^ memory B cells, CD24^++^CD38^++^ transitional B cells, CD24^−^CD38^++^ plasmablasts, CD21^−/low^CD38^−/low^ B cells and CD23^+^ B cells,^64^ as shown in Figure S1. The number of gated events was used to determine the respective B-cell subpopulation frequencies.

### Measurement of mature microRNAs in B cells

The integrity of the B-cell RNA samples was checked using an Agilent Bioanalyzer 2100 with RNA 6000 Pico kits (Agilent Technologies). RNA concentrations were measured using a NanoDrop ND-1000 Spectrophotometer (Thermo Fisher Scientific).

After the RNA quality check, the levels of mature miRNAs and miRNA isoforms were measured by quantitative polymerase chain reaction (qPCR) using custom Specialty TaqMan Array Cards (48 format) for standard TaqMan miRNA assays with FAM-labelled probes (Thermo Fisher Scientific). Reverse transcription (RT) was performed using 35 ng of RNA per sample and a custom target-specific stem-loop RT primer pool. The cDNA was then preamplified in 12 PCR cycles with custom PreAmp primers following the manufacturer's protocol for the use of Megaplex primer pools (Applied Biosystems). Finally, the qPCR measurements were prepared using TaqMan Universal Master Mix II with UNG and performed using a ViiA 7 Real-Time PCR System (Applied Biosystems) with 45 amplification cycles. Each miRNA was measured either in single wells or in technical triplicates, and hsa-miR-191-5p was included in the analysis as reference miRNA.^65^

Raw *C*_T_ values were determined using the ExpressionSuite software version 1.3 (Thermo Fisher Scientific) and averaged over replicates. Assays for which the automatic threshold algorithm failed were excluded from the further analyses. Missing values, which resulted when no *C*_T_ value could be determined within 45 PCR cycles, were imputed using the expectation-maximisation algorithm that is implemented in the R package nondetects.^66^ For sensitivity analyses, the imputed dataset was truncated to a maximum *C*_T_ value of 35. The data were normalised to the reference miRNA (Δ*C*_T_ method) and converted to the linear scale using the equation 2^−Δ^*^C^*^T^×1,000.

### Genotyping

The DNA extracted from the blood samples was used to determine the genotypes of MIR SNPs and tagging SNPs for HLA-A*02:01^67^ and HLA-DRB1*15:01^68^ for each subject. The genotyping was done by PCR with allele-specific TaqMan probes labelled with FAM or VIC on custom Specialty TaqMan Array Cards (Thermo Fisher Scientific). For each reaction, we used ∼2 ng of DNA that was diluted in TaqMan Universal Master Mix II with UNG (Applied Biosystems). PCR amplification was carried out following manufacturer's instructions in a ViiA 7 Real-Time PCR System (Applied Biosystems). Genotype calling was performed by allelic discrimination with the TaqMan Genotyper Software version 1.6 (Applied Biosystems).

### Evaluation of primary microRNA processing efficiencies

We employed a luciferase reporter system to evaluate whether the pri-miRNA processing efficiency depends on the genotype of MS-associated SNPs (Figure 1). The assay has been adapted from Allegra et al.^69^^,^^70^ and is based on a luciferase reporter gene that is fused with a miRNA precursor sequence. We first tested the assay with the pri-miRNA hsa-mir-16-1 because its processing has already been extensively studied.^44^^,^^71^^,^^72^

We utilised the luciferase reporter vector CmiT000001-MT05 (GeneCopoeia), which encodes the secreted *Gaussia* luciferase (GLuc) as reporter gene and the secreted alkaline phosphatase (SEAP) as internal control for normalisation. In addition, we purchased miExpress precursor miRNA expression plasmids for the prioritised miRNAs as well as for hsa-mir-16-1 from GeneCopoeia. The plasmids were amplified in *E. coli* cultures and purified using the Qiagen EndoFree Plasmid Maxi Kit and QIAfilter Midi Cartridges.

Mutations resembling the naturally occurring genetic variants in the miRNA-coding sequence region (*i.e.*, MIR SNPs) were introduced into the precursor miRNA plasmids. In the hsa-mir-16-1 precursor plasmid, an artificial C>T mutation at position 3’ (+19) was introduced. This mutation has been previously shown to suppress the cleavage of hsa-mir-16-1 by the Microprocessor complex.^44^^,^^73^ The primers for the desired point mutations were designed using the QuikChange Primer Design program from Agilent Technologies and purchased from Thermo Fisher Scientific. The mutageneses were performed using the QuikChange Lightning Site-Directed Mutagenesis Kit (Agilent Technologies) according to the manufacturer's instructions. The success of each mutagenesis was confirmed by commercial Sanger sequencing using the EGFP-C-for primer (Microsynth Seqlab).

The different miRNA precursor sequences with the respective alleles were cloned into the luciferase reporter vector at the 3’ untranslated region (UTR) of the GLuc gene. This was done by conventional restriction digestion and ligation. *Sfa*AI and either *Eco*RI or *Xho*I (Thermo Fisher Scientific) were used as restriction enzymes. The precursor miRNA fragments were prepared with the NucleoSpin Gel and PCR Clean-up Kit (Macherey-Nagel) and ligated into the restriction sites of the luciferase reporter vector with T4 DNA ligase (Thermo Fisher Scientific). Successful cloning was confirmed by gel electrophoresis or DNA sequencing (Microsynth Seqlab). The cloned constructs were then amplified in *E. coli* and purified using the Nucleobond Xtra Midi EF Kit (Macherey-Nagel).

HeLa cells were transiently transfected with the reporter constructs in 3 biological replicates using the FuGENE HD Transfection Reagent (Promega). The HeLa cells were originally obtained from the German Cancer Research Center (Heidelberg, Germany). They were regularly tested for mycoplasma contamination using the MycoAlert Mycoplasma Detection Kit (Lonza). For establishing the assay with the hsa-mir-16-1 constructs, four different amounts of plasmid DNA (100 ng, 200 ng, 300 ng and 500 ng) were used, and supernatants were collected at 24 h, 48 h and 72 h. Otherwise, 200 ng and 300 ng of plasmid DNA were used, and the transfected HeLa cells were cultured for 24 h and 48 h. In this time, the stem-loop structure that is formed by the GLuc-precursor miRNA hybrid transcript can be recognised by the endogenous Microprocessor complex. A more efficient cleavage of the miRNA stem-loop leads to a preferential degradation of the luciferase transcript and thus reduced reporter protein levels. The GLuc and SEAP activities were measured in technical duplicates using the Secrete-Pair Dual Luminescence Assay Kit (GeneCopoeia) and a GloMax-Multi Detection System (Promega) according to the manufacturers’ recommendations.

Afterwards, average ratios of the luminescence catalysed by GLuc and SEAP were calculated. A higher GLuc/SEAP ratio indicates a lower Drosha cleavage activity. The ratios were further normalised to those resulting for the negative control, that is, the original luciferase reporter vector without an inserted miRNA precursor fragment. The decrease in relative luminescence as compared to the negative control is a measure of the pri-miRNA processing efficiency.^70^ In this calculation, the processing efficiency for the negative control vector is set to zero.

### Target gene analysis

We aimed to identify direct target genes of those miRNAs that were found to be processed dependent on the genotype of MS-associated SNPs. For this purpose, we conducted a transcriptome profiling analysis of HeLa cell cultures (Figure 1). The cells were transiently transfected with the respective miExpress precursor miRNA expression plasmids (GeneCopoeia) or the scrambled control clone for the pEZX-MR04 vector (CmiR0001-MR04, GeneCopoeia) as negative control using the FuGENE HD Transfection Reagent (Promega). Total RNA was isolated 24 h (from 2 replicates) and 48 h (from 4 replicates) following transfection using the miRNeasy Mini Kit (Qiagen). RNA concentrations were measured using a NanoDrop ND-1000 Spectrophotometer (Thermo Fisher Scientific), and RNA integrity was assessed with an Agilent 2100 Bioanalyzer using RNA 6000 Nano kits (Agilent Technologies).

To confirm the overexpression of mature miRNA molecules, a qPCR analysis was performed with predesigned TaqMan single-tube assays from Thermo Fisher Scientific. The cDNA was generated from 10 ng intact RNA with miRNA-specific stem-loop primers and mixed with TaqMan Universal Master Mix II with UNG (Applied Biosystems). The qPCR measurement was then run in triplicates for 45 cycles on a ViiA 7 Real-Time PCR System (Applied Biosystems). As reference miRNA, hsa-miR-191-5p was chosen.^65^ The ExpressionSuite software version 1.3 (Thermo Fisher Scientific) was used to determine *C*_T_ values based on the FAM reporter signals. The average *C*_T_ values per triplicate were normalised to those of the reference miRNA to obtain Δ*C*_T_ values.

The same RNA samples were used for the transcriptome analysis. From each sample, 210 ng of total RNA was used as starting material to generate amplified, fragmented and biotinylated single-stranded sense strand DNA using the GeneChip WT PLUS Reagent Kit (Applied Biosystems). The hybridisation on high-resolution Clariom D arrays for human (Applied Biosystems) was then carried out for 16 h at 45°C in a GeneChip Hybridization Oven 645 (Affymetrix) following the manufacturer's protocol. After washing and staining in a GeneChip Fluidics Station 450 (Affymetrix), the microarrays were scanned using a GeneChip Scanner 3000 7G (Affymetrix). The scans were imported to the Affymetrix GeneChip Command Console version 4.0 to extract the signal intensities for the >6.7 million 25mer oligonucleotide probes per array. The Transcriptome Analysis Console (TAC) version 4.0.2 (Applied Biosystems) was used for initial quality control assessments and for processing the data with the signal space transformation robust multi-array average algorithm for background adjustment, quantile normalisation, probe set summarisation and log2 transformation.

Based on the processed data, an analysis of differential gene expression was performed in TAC. Potential target genes of the miRNAs were identified by filtering those transcript clusters (TC) that were significantly lower expressed at both time points (*i.e.*, at 24 h and at 48 h), with at least 33.3% lower levels at one time point, in HeLa cells transfected with the precursor miRNA plasmids compared to HeLa cells transfected with the negative control plasmid. A nominal significance level of α=5% was chosen. The filtering result was then further confined to protein-coding genes with an official gene symbol according to the HGNC database.^74^ Finally, to obtain the most likely direct target genes, we used RNAhybrid version 2.2^75^ for checking whether there are predicted binding sites for the canonical miRNAs or isomiRs in the 3’ UTRs of the mRNAs. The 3’ UTR sequences were retrieved with the BioMart tool from Ensembl release 101,^76^ and we requested an mfe of less than −16 kcal/mol for the miRNA-target interaction as well as a perfect match with the seed region of the miRNA (*i.e.*, position 2 to 7).

We evaluated whether the genes were already listed as predicted or experimentally determined target genes in the miRWalk 3.0 database^77^ and in miRTarBase release 8.0,^78^ respectively. The search in miRWalk was restricted to 3’ UTR miRNA binding sites. Moreover, to assign the putative target genes to functional categories, we searched them in signalling pathways using Reactome release 75,^79^ in Gene Ontology sets using Enrichr^80^ and in the UniProt Knowledgebase.^81^ The categories with the greatest gene set overlap were recorded. We also studied whether or not the genes are expressed in cell types of the blood and brain using the microarray data from Novershtern et al.^82^ (GEO accession GSE24759) and the RNA-sequencing data from Darmanis et al.^83^ and by demanding a transcript level of >100.

### Statistical analyses

The statistical analyses were performed in R version 3.6.2 unless stated otherwise. For descriptive statistics, means and standard deviations (SD) per group were calculated. The inferential statistics were exploratory in nature. Therefore, the significance level was generally set at α=0.05 to minimise the risk of type II errors. The data were visualised in beeswarm/violin plots and bar plots using the R packages beeswarm, vioplot and gplots, respectively.

We tested for differences in B-cell subpopulation frequencies and miRNA expression levels (in linear scale) between the study groups using linear models while adjusting for age and sex as potential confounding variables. Type II *F*-tests were calculated for all main effects in the models with the R package car.^84^ Pairwise comparisons between the study groups were performed with Tukey post hoc tests adjusted for age and sex using the R package multcomp.^85^ We used Pearson correlation tests to compare the levels of canonical miRNAs and their potential isomiRs.

The SNP genotype distributions were compared between the MS patients and healthy controls using one-tailed Fisher's exact tests in order to evaluate the association of risk alleles with MS in our study population. Additionally, allelic ORs were calculated using the Wald method. The effect of each MIR SNP on the expression level of the respective mature miRNA was examined in *cis-*miR-eQTL analyses. For this purpose, we fitted linear mixed-effects models using the R package lme4.^86^ The study group was treated as a random effect. Genotype, age and sex were considered as fixed effects. The models were fitted without interaction term and with the genotype effect being captured in an additive fashion using the restricted maximum likelihood approach. Significance values were then computed with type II Wald χ^2^ tests using the R package car.^84^ For pairwise genotype comparisons, Tukey post hoc tests adjusted for age, sex and group were carried out.

The balanced dataset from the luciferase reporter assays was analysed using an additive 3-way analysis of variance (ANOVA), with amount of plasmid DNA, time point and genotype as independent variables and GLuc/SEAP ratios or the decrease in relative luminescence as dependent variable. Accordingly, *F*-tests were used to examine the significance of individual effects. Welch two-tailed *t*-tests were applied for comparing the data that were obtained under the same experimental conditions with plasmids, which represent the two alleles of a particular SNP.

### Ethics

This study was conducted with approvals by the local ethics committee of the University of Rostock (permit numbers A 2014-0112 and A 2016-0188) and in compliance with the principles of the Declaration of Helsinki. All subjects gave prior written informed consent for the scientific use of their biosamples.

### Role of funders

The funders had no role in the study design, data collection, analysis and interpretation, decision to publish or preparation of the manuscript.

## Results

### Prioritised microRNAs from genetic risk loci for MS

We identified a total of 11 pri-miRNAs from MS-associated genetic regions that were considered for the further investigations in the present study (Table 1). These pri-miRNAs contain SNPs in the adjacent 50 bp around the miRNA precursor sequence that were suspected to influence the Drosha-mediated enzymatic cleavage of the stem-loop. In case of hsa-mir-4304, two such MIR SNPs were found. Seven of the 12 ascertained MIR SNPs are in complete LD (*D’*=1) with the nearby MS lead SNP from the GWAS,^26^ which means that one MIR SNP allele is always inherited together with one specific MS lead SNP allele according to the 1000 Genomes haplotype data.^52^ Six of the 12 MIR SNPs are rare genetic variants with a global MAF of less than 2% (Table 1).

**Table 1:** Prioritised microRNAs from MS-associated genetic regions.

Primary microRNA	miRBase accession	Mature forms	Chromosome (strand)	Gene locus	MS lead SNP identifier a	MIR SNP identifier	SNP distance in bp b	LD *D'* (EUR)	MIR SNP allele frequency b	MIR SNP position	MIR SNP location
hsa-mir-26a-2	MI0000750	5p and 3p	chr12 (‒)	*CTDSP2*	rs701006	rs41292017	111546	1.0000	G: 98.2%; A: 1.8%	3′ (+21)	CNNC
hsa-mir-199a-1	MI0000242	5p and 3p	chr19 (‒)		rs12609500	rs1005039	245847	1.0000	C: 98.6%; T: 1.4%	3′ (+24)	CNNC
hsa-mir-548ac	MI0016762	3p	chr1 (‒)	*CD58*	rs10801908	rs1414273	12156	0.9916	C: 57.2%; T: 42.8%	3′ (+11)	basal stem
hsa-mir-934	MI0005756	5p	chrX (+)	*VGLL1*	rs2807267	rs73558572	33408	0.9674 c	G: 99.0%; A: 1.0%	5′ (‒6)	basal stem
hsa-mir-3661	MI0016062	5p	chr5 (+)		rs244656	rs779704489	111732	0.7328 d	A: 98.1%; C: 1.9%	3′ (+34)	flanking region
hsa-mir-3671	MI0016072	3p	chr1 (‒)	*JAK1*	rs72922276	rs521188	94200	1.0000	A: 89.3%; G: 10.7%	5′ (‒6)	basal stem
hsa-mir-4252	MI0015864	3p	chr1 (‒)		rs2986736	rs2003071	22681	0.7869	C: 91.1%; T: 8.9%	3′ (+38)	flanking region
hsa-mir-4304	MI0015832	5p	chr12 (‒)	*PITPNM2*	rs7975763	rs78351440, rs77905262	108764, 108773	1.0000, 1.0000	C: 94.0%; A: 6.0%, G: 89.3%; C: 10.7%	5′ (‒24), 5′ (‒15)	flanking region, flanking region
hsa-mir-4423	MI0016760	5p and 3p	chr1 (+)		rs35486093	rs817478	130257	0.7445	A: 73.2%; C: 26.8%	3′ (+18)	CNNC
hsa-mir-4464	MI0016812	5p	chr6 (+)	*AL132996.1*	rs72928038	rs77896647	45799	1.0000 c	G: 98.9%; A: 1.1%	3′ (+22)	flanking region
hsa-mir-4492	MI0016854	3p	chr11 (+)		rs12365699, rs6589706	rs7926599	38244, 33717	1.0000, 1.0000	G: 98.5%; C: 1.5%	3′ (+40)	flanking region

Eleven miRNA-coding regions were identified in the vicinity of MS-associated SNPs. A total of 12 SNPs are located in these sequence regions and are in LD with the MS lead SNPs. These MIR SNPs were suspected to affect the Drosha-mediated processing of the miRNA stem-loop sequences. For three of the primary miRNAs, two mature miRNA molecules (from the 5′ and 3′ arm) are annotated in the miRBase database.^36^ Six of the miRNA loci overlap with intronic gene regions. The position of the MIR SNPs relative to the predicted Drosha cleavage sites is indicated. Three of the MIR SNPs modify a downstream CNNC motif. bp=base pairs, EUR=European, LD=linkage disequilibrium, MIR/miRNA=microRNA, MS=multiple sclerosis, SNP=single-nucleotide polymorphism.

a taken from the latest genome-wide association study in MS.^26^

b distances and global frequencies of the forward strand alleles are given according to dbSNP build 151 and the GRCh38 reference genome assembly.

c the LDpair tool^51^ did not provide LD measures for the European population, thus r2 and D' are provided for the whole 1000 Genomes panel.^52^

d no information for the MIR SNP in LDpair,^51^ thus SNP rs3895755 served as proxy.

Except for hsa-mir-26a-2 and hsa-mir-199a-1, all selected pri-miRNAs are primate-specific,^36^ and all were classified as Drosha-dependent in the study by Kim et al.^87^ Six of the miRNA stem-loops are presumably processed from introns of host gene transcripts (Table 1). Our research group has previously explored the role of hsa-mir-548ac, which is encoded in the first intron of the *CD58* gene, and we found that MS risk allele carriers have increased levels of the mature miRNA in blood-derived cells.^48^^,^^49^ Another prioritised pri-miRNA is hsa-mir-934, which is located in the fourth intron of the *VGLL1* gene, the only sex chromosomal MS risk locus that has been reported in the GWAS from 2019.^26^ The majority of the selected miRNAs (n=7) have a 4-digit number. This means that they were discovered relatively recently, that they are usually not covered by standard qPCR- or array-based miRNA measurement platforms, and that their regulatory functions are often still largely unclear.

Three of the MIR SNPs affect the basal stem (Table 1). For instance, the A allele of SNP rs73558572 causes a mismatch in the hsa-mir-934 secondary structure. All the other MIR SNPs are located in the flanking segments. Three of them belong to a downstream CNNC motif (rs817478, rs1005039 and rs41292017). We hypothesised that the pri-miRNA processing efficiency and/or the Drosha cleavage sites are dependent on the allele status of these SNPs.

### Characteristics of the study population and samples

The study population comprised 91 subjects in total. From these, 120 blood samples were collected and considered eligible for the analyses in the present study. The study population was divided into 6 study groups: healthy controls (n=28), patients with PPMS who were treated with glucocorticosteroid pulse therapy (n=13) and four groups of RRMS patients (n=50) that were differentiated by the type of treatment received (Table 2). A subset of 29 RRMS patients was treated with natalizumab. Seven RRMS patients were treated with fingolimod (n=3), glatiramer acetate (n=3) or interferon-β-1b (n=1) before switching to an IRT (RRMS before IRT group). These patients received a first treatment course of intravenous alemtuzumab (n=4) or oral cladribine (n=3) immediately after the blood collection, and they all donated at least one additional blood sample at a later time point during therapy. The samples from patients under therapy with alemtuzumab (n=37) or cladribine (n=6) were obtained at least 4 months after the last treatment week, *i.e.*, after partial B-cell repopulation following B-cell depletion. In the alemtuzumab group, one to four blood samples were collected per patient.

**Table 2:** Basic characteristics of the study groups.

Study group	Samples, n	Subjects, n	Women, n	Men, n	Age in years, mean ± SD	Duration of disease in years, mean ± SD	Relapses in previous year, mean ± SD	EDSS score, mean ± SD (mv)
Healthy controls	28	28	17	11	28.0 ± 8.9	—	—	—
MS patients	92	63	38	25	39.4 ± 13.1	8.3 ± 6.6	0.3 ± 0.7	3.0 ± 1.6 (10)
– PPMS	13	13	5	8	58.7 ± 9.8	9.7 ± 4.6	0.0 ± 0.0	4.9 ± 1.7
– RRMS, natalizumab	29	29	16	13	44.4 ± 9.0	14.0 ± 6.1	0.0 ± 0.0	3.2 ± 1.7 (8)
– RRMS, before IRT	7	7	5	2	33.7 ± 12.2	2.8 ± 2.8	1.1 ± 1.2	2.1 ± 0.8
– RRMS, alemtuzumab	37	15	12	3	29.2 ± 4.8	4.2 ± 3.5	0.5 ± 0.7	2.4 ± 1.0 (1)
– RRMS, cladribine	6	6	5	1	42.0 ± 12.5	8.9 ± 9.0	0.5 ± 0.8	2.5 ± 0.8 (1)

A total of 120 blood samples from 91 subjects were analysed in this study. Up to four blood samples were collected per patient. From the 7 patients that provided a blood sample immediately before the initiation of IRT, at least one sample was also obtained after the administration of alemtuzumab (n=4) or cladribine (n=3). Age and clinical characteristics are reported for the time point of blood collection. For 10 samples, no information about the patients' current degree of disability (as rated by the EDSS^60^) was available, and thus the means and SDs refer to cases with valid data. — = not available, EDSS=Expanded Disability Status Scale, IRT=immune reconstitution therapy, MS=multiple sclerosis, mv=missing values, n=number, PPMS=primary progressive multiple sclerosis, RRMS=relapsing-remitting multiple sclerosis, SD=standard deviation.

There was a female preponderance in the healthy control group and in the RRMS groups but not in the PPMS group (Table 2). The average age of the controls was 28.0 years. The patients were older, with a mean age of 39.4 years and a mean disease duration of 8.3 years. They had, on average, 0.3 relapses in the year prior to the blood sampling and a mean EDSS score of 3.0. The patients with PPMS had the highest age and the highest degree of disability on average. The patients who were about to switch to an IRT were in an early phase of the disease, and they had the highest relapse rate. To account for these differences, we controlled for potential confounding by age, sex and group assignment in the statistical analyses of the data described hereafter.

The number of cells after enrichment of B cells averaged 4.3 million. Flow cytometry of the freshly isolated cells revealed a mean B-cell purity of 85.2% (2 out of 120 samples had missing values). Using the cryopreserved B cells, we were able to distinguish 8 B-cell subpopulations based on the expression of cell surface markers. The mean percentages of the different B-cell subsets across the 6 study groups are shown in Table S1. In general, CD27^−^IgD^+^ naive B cells accounted for the largest proportion (up to 72.4% on average), whereas transitional and CD21^−/low^CD38^−/low^ B cells occurred at the lowest frequencies (up to 7.4% and 9.0% on average, respectively). When comparing the 6 study groups, there were significant differences in the proportions of all subpopulations (*F*-test P<0.05). For instance, CD27^+^IgD^+^ non-switched memory B cells represented ∼24% of CD19^+^ cells in the healthy controls. The respective percentages were higher, on average, in RRMS patients treated with natalizumab (43.0%) and much lower in RRMS patients on IRT with alemtuzumab or cladribine (up to 8.9% on average). Age and sex did not explain the variance in these data (*F*-test P>0.05).

### Differential expression of mature microRNAs in B cells

According to miRBase,^36^ 14 mature miRNAs are produced from the prioritised pri-miRNAs from MS-associated genetic loci (Table 1). Standard TaqMan assays were available for 13 of these. The qPCR assay design failed for hsa-miR-4492-3p due to a high GC content of 94%. To consider the possible formation of different mature miRNAs from the same stem-loop, we utilised information from IsomiR Bank^54^ and miRCarta^55^ to define potential 3’ isomiRs (n=7), 5’ and 3’ isomiRs (n=2) and putative miRNAs that may be generated from the other arm than annotated in miRBase^36^ (n=6). Thus, a total of 28 assays were used for the measurement of those miRNAs in B cells from MS patients and healthy controls.

The data of 22 qPCR assays could be included in the further analyses. For each study group, the average expression levels relative to the reference miRNA hsa-miR-191-5p, which could be detected with *C*_T_ values <20 in all samples, are given in Table 3. For the other 6 assays, we did not obtain valid data as the automatic threshold algorithm failed (THOLDFAIL flag): hsa-miR-934-5p|{isomiR}|14_33|, hsa-miR-3661-5p|{hsa-miR-3661}|20_41|, hsa-miR-3661-5p|{isomiR}|20_40|, hsa-miR-3671-5p|{isomiR}|3_19|, hsa-miR-3671-5p|{isomiR}|3_19|sub.6.T>C and hsa-miR-4252-3p|{hsa-miR-4252}|34_52|.

**Table 3:** Relative expression of mature microRNAs in B cells from MS patients and controls.

MicroRNA assay a	MicroRNA sequence	Detection rate b, %	Healthy controls (n=28), mean ± SD (mv c)	PPMS (n=13), mean ± SD (mv c)	RRMS, natalizumab (n=29), mean ± SD (mv c)	RRMS, before IRT (n=7), mean ± SD (mv c)	RRMS, alemtuzumab (n=37), mean ± SD (mv c)	RRMS,cladribine (n=6), mean ± SD (mv c)	P-value d
hsa-miR-26a-2-5p|{hsa-miR-26a-5p}|13_34|	UUCAAGUAAUCCAGGAUAGGCU	100.0	2.06 ± 0.35	1.99 ± 0.55	1.76 ± 0.43	1.98 ± 0.29	2.16 ± 0.44	2.27 ± 0.27	**0.0014**
hsa-miR-26a-2-5p|{isomiR}|13_33|	UUCAAGUAAUCCAGGAUAGGC	100.0	0.69 ± 0.43	0.58 ± 0.67	0.36 ± 0.46	0.70 ± 0.49	0.87 ± 0.56	0.77 ± 0.08	**0.0011**
hsa-miR-26a-2-3p|{hsa-miR-26a-2-3p}|51_72|	CCUAUUCUUGAUUACUUGUUUC	91.7	13.32 ± 1.56 (1)	13.97 ± 1.07 (5)	13.31 ± 0.99 (1)	14.33 ± 1.39	13.56 ± 0.80 (2)	13.18 ± 0.89 (1)	**0.0418**
hsa-miR-199a-1-5p|{hsa-miR-199a-5p}|5_27|	CCCAGUGUUCAGACUACCUGUUC	50.8	21.20 ± 5.30 (15)	20.76 ± 5.40 (7)	25.30 ± 5.32 (20)	21.12 ± 6.28 (4)	17.79 ± 4.58 (11)	18.28 ± 4.62 (2)	**0.0177**
hsa-miR-199a-1-5p|{isomiR}|5_26|	CCCAGUGUUCAGACUACCUGUU	98.3	12.12 ± 2.27	12.38 ± 1.97 (1)	12.59 ± 1.64	10.42 ± 1.88	10.20 ± 2.16 (1)	12.45 ± 3.24	**0.0005**
hsa-miR-199a-1-3p|{hsa-miR-199a-3p}|46_67|	ACAGUAGUCUGCACAUUGGUUA	100.0	7.98 ± 1.54	8.12 ± 1.43	8.87 ± 1.25	6.86 ± 1.83	6.71 ± 2.03	7.72 ± 1.89	**0.0006**
hsa-miR-199a-1-3p|{isomiR}|46_66|	ACAGUAGUCUGCACAUUGGUU	100.0	8.15 ± 1.25	8.24 ± 0.99	8.75 ± 0.88	7.43 ± 1.60	7.17 ± 1.74	8.11 ± 1.76	**0.0008**
hsa-miR-548ac-5p|{isomiR}|16_36|	AAAGUUAUUGUGGUUUUUGCU	13.3	29.92 ± 5.06 (22)	44.07 ± 0.95 (13)	34.57 ± 4.77 (27)	31.00 ± 6.02 (6)	30.58 ± 5.79 (31)	28.28 ± 6.14 (5)	0.8165
hsa-miR-548ac-3p|{hsa-miR-548ac}|52_73|	CAAAAACCGGCAAUUACUUUUG	30.0	25.95 ± 5.32 (21)	28.16 ± 4.72 (11)	22.94 ± 5.08 (15)	25.46 ± 5.82 (5)	25.43 ± 5.38 (26)	40.48 ± 1.20 (6)	0.8473
hsa-miR-934-5p|{hsa-miR-934}|14_35|	UGUCUACUACUGGAGACACUGG	12.5	29.39 ± 5.61 (23)	42.23 ± 0.95 (13)	31.75 ± 5.09 (26)	29.80 ± 5.90 (6)	30.56 ± 4.46 (31)	41.49 ± 1.20 (6)	0.6109
hsa-miR-934-3p|{isomiR}|50_71|	GAGUCUCCAGUAAUGGACGGGA	18.3	27.58 ± 4.82 (21)	31.40 ± 4.31 (12)	30.16 ± 4.91 (25)	29.34 ± 4.65 (6)	29.21 ± 4.84 (30)	25.64 ± 4.74 (4)	0.9388
hsa-miR-3671-3p|{hsa-miR-3671}|59_80|	AUCAAAUAAGGACUAGUCUGCA	13.3	33.75 ± 1.42 (27)	37.04 ± 0.95 (13)	31.33 ± 2.71 (27)	23.75 ± 4.79 (4)	27.03 ± 4.29 (29)	24.11 ± 4.73 (4)	0.1381
hsa-miR-4304-5p|{hsa-miR-4304}|10_26|	CCGGCAUGUCCAGGGCA	100.0	10.72 ± 0.72	10.12 ± 0.98	10.84 ± 0.57	10.63 ± 0.81	10.92 ± 0.84	9.27 ± 1.13	**3.3**×10^−^**^5^**
hsa-miR-4304-3p|{isomiR}|35_52|	GCUCUGUGACUGCUGCCA	100.0	10.74 ± 0.72	10.38 ± 1.17	11.69 ± 0.82	10.84 ± 0.98	11.01 ± 0.86	9.84 ± 0.82	**8.2**×10^−^**^5^**
hsa-miR-4423-5p|{hsa-miR-4423-5p}|12_33|	AGUUGCCUUUUUGUUCCCAUGC	54.2	21.19 ± 4.59 (13)	23.50 ± 4.57 (9)	19.25 ± 3.64 (7)	24.88 ± 4.68 (3)	22.35 ± 4.69 (21)	20.21 ± 4.60 (2)	0.9757
hsa-miR-4423-5p|{isomiR}|12_32|	AGUUGCCUUUUUGUUCCCAUG	57.5	19.69 ± 3.88 (11)	21.69 ± 4.49 (8)	18.49 ± 3.34 (5)	21.76 ± 4.65 (4)	21.51 ± 3.58 (18)	25.73 ± 2.64 (5)	0.0652
hsa-miR-4423-3p|{hsa-miR-4423-3p}|48_68|	AUAGGCACCAAAAAGCAACAA	100.0	13.40 ± 1.00	13.09 ± 1.57	13.56 ± 0.85	13.57 ± 1.00	13.52 ± 0.95	12.64 ± 0.67	**0.0413**
hsa-miR-4423-3p|{isomiR}|48_67|	AUAGGCACCAAAAAGCAACA	100.0	13.03 ± 0.99	12.53 ± 1.09	13.30 ± 0.65	13.21 ± 0.97	13.14 ± 0.96	12.63 ± 0.71	**0.0227**
hsa-miR-4464-5p|{hsa-miR-4464}|11_31|	AAGGUUUGGAUAGAUGCAAUA	100.0	15.91 ± 1.29	15.11 ± 1.06	16.30 ± 1.04	16.55 ± 1.36	15.88 ± 0.97	14.55 ± 1.00	**0.0063**
hsa-miR-4464-5p|{isomiR}|15_35|	UUUGGAUAGAUGCAAUAAAGU	71.7	18.66 ± 3.73 (9)	20.00 ± 4.20 (7)	18.24 ± 3.82 (7)	16.71 ± 2.52 (1)	17.52 ± 3.70 (7)	20.73 ± 3.99 (3)	0.8573
hsa-miR-4464-3p|{isomiR}|61_79|	UUAUUGCAUCUAUCCAAAC	5.8	42.23 ± 0.94 (28)	29.56 ± 5.65 (11)	32.30 ± 5.21 (26)	42.45 ± 0.81 (7)	34.71 ± 4.21 (35)	41.01 ± 1.20 (6)	0.8342
hsa-miR-4492-3p|{isomiR}|58_74|	GGGCUGGGCGCGCGCCA	100.0	6.34 ± 1.26	5.85 ± 1.27	7.14 ± 1.10	7.01 ± 0.89	6.84 ± 0.98	5.24 ± 0.79	**0.0006**

A total of 120 B-cell samples were analysed, and 22 qPCR assays gave valid data. For 6 primary miRNAs, at least one mature miRNA molecule (from the 5′ or 3′ arm) could be detected in at least half of the samples. Average Δ*C*_T_ values are reported as a measure of the miRNA expression relative to hsa-miR-191-5p (reference miRNA) for each of the 6 study groups. Lower Δ*C*_T_ values correspond to higher expression levels. The P-values indicate whether there is a difference in the level of miRNA expression between the groups. Significant differences (P<0.05) are marked in bold. IRT=immune reconstitution therapy, miRNA=microRNA, MS=multiple sclerosis, mv=missing values, n=number, PPMS=primary progressive multiple sclerosis, qPCR=quantitative polymerase chain reaction, RRMS=relapsing-remitting multiple sclerosis, SD=standard deviation.

a the miRNAs are specified following the nomenclature by Cloonan et al.^56^

b the percentage of samples for which a *C*_T_ value could be determined within 45 qPCR cycles.

c the number of samples in which the miRNA could not be detected and for which *C*_T_ values were thus imputed.

d *F*-test P-values for linear models fitted to the data in linear scale and adjusted for age and sex.

Six miRNAs were detected in less than half of the samples: hsa-miR-548ac-5p and -3p, hsa-miR-934-5p and -3p, hsa-miR-3671-3p and hsa-miR-4464-3p. The highest expression levels (mean Δ*C*_T_ values <10) were seen for hsa-miR-26a-5p and hsa-miR-199a-3p isoforms as well as for hsa-miR-4492-3p|{isomiR}|58_74|. In those cases where both the canonical miRNA and an isomiR from the same arm of the stem-loop were measured, the data were highly correlated (Pearson correlation P≤0.0005). This was also true for the two forms of hsa-miR-4464-5p that are shifted by 4 nt. Therefore, only one form was considered for the visualisation of the qPCR data. However, it should be noted that the levels of the 3’ isomiR of hsa-miR-199a-5p were much higher than those of the corresponding canonical miRNA (mean Δ*C*_T_ 11.58 vs. 20.94). Moreover, only one mature miRNA is recorded in miRBase^36^ for hsa-mir-548ac, hsa-mir-934, hsa-mir-4304 and hsa-mir-4464, but we could measure miRNA molecules derived from both the 5’ arm and the 3’ arm.

**Figure 2:**
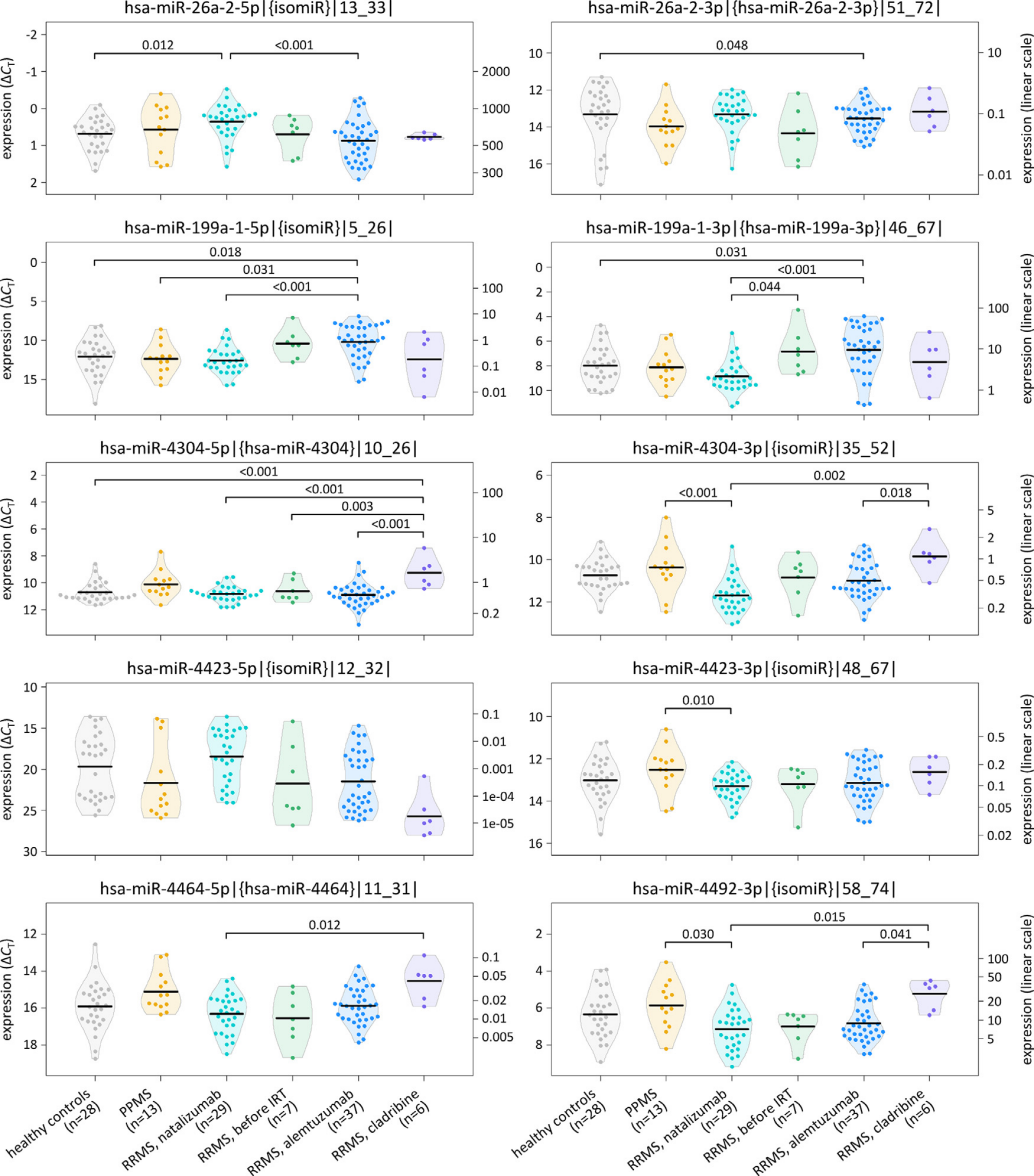
Differential expression of microRNAs in B cells from MS patients and healthy controls. The expression of 10 mature miRNAs and isomiRs is visualised for the six study groups. These miRNAs could be detected in >50% of the samples and are derived from 6 primary miRNA transcripts from MS-associated genetic regions. For this plot, we always selected the 5p and 3p miRNA forms (canonical miRNA or isomiR) that had the lowest P*-*value in the group comparisons (Table 3). The expression was quantified relative to the reference miRNA hsa-miR-191-5p. Higher data points indicate higher expression levels. The y-axis on the left displays Δ*C*_T_ values in an inverted manner and the y-axis on the right displays the data converted in linear scale (2^−Δ^*^C^*^T^×1000). Black horizontal lines indicate the means per group. Significance values <0.05 from pairwise Tukey post hoc tests are shown above the brackets. IRT=immune reconstitution therapy, miRNA=microRNA, MS=multiple sclerosis, n=number, PPMS=primary progressive multiple sclerosis, RRMS=relapsing-remitting multiple sclerosis.

In comparison of the study groups, significant expression differences were found for 7 canonical miRNAs and 6 isomiRs (*F*-test P<0.05) (Table 3). These differences were also significant in the sensitivity analysis (*i.e.*, after replacing *C*_T_ values >35 by a value of 35). The differentially expressed mature miRNAs originate from 6 pri-miRNAs: hsa-mir-26a-2, hsa-mir-199a-1, hsa-mir-4304, hsa-mir-4423, hsa-mir-4464 and hsa-mir-4492 (Figure 2). Compared with the healthy controls, the levels of hsa-miR-26a-2-3p were significantly lower in RRMS patients treated with alemtuzumab (Tukey's test P=0.048). In contrast, the levels of hsa-miR-199a-5p and -3p were higher in alemtuzumab-treated cases than in the controls. Significantly higher levels of hsa-miR-4304-5p were measured in RRMS patients receiving cladribine in comparison to the healthy group and all other RRMS groups (Tukey's test P≤0.003). In the B-cell samples of the cladribine-treated patient group, we also found, on average, the highest levels of hsa-miR-4464-5p and a 1 nt shifted isomiR of hsa-miR-4492-3p. The average expression of hsa-miR-4423-3p|{isomiR}|48_67| was highest in patients with PPMS.

### Association of genetic variants with microRNA expression

The 12 MIR SNPs as well as two HLA allele tagging SNPs were genotyped for all subjects (Table S2). Overall, the allele frequencies in the study population roughly corresponded to the global allele frequencies (Table 1). We could confirm a significant association between the risk allele of SNP rs1414273 (OR=2.52) as well as the HLA-DRB1*15:01 allele (OR=2.69) and MS (Fisher's test P<0.05). However, for 4 MIR SNPs, we did not detect the rare allele in either MS patients or controls.

**Figure 3:**
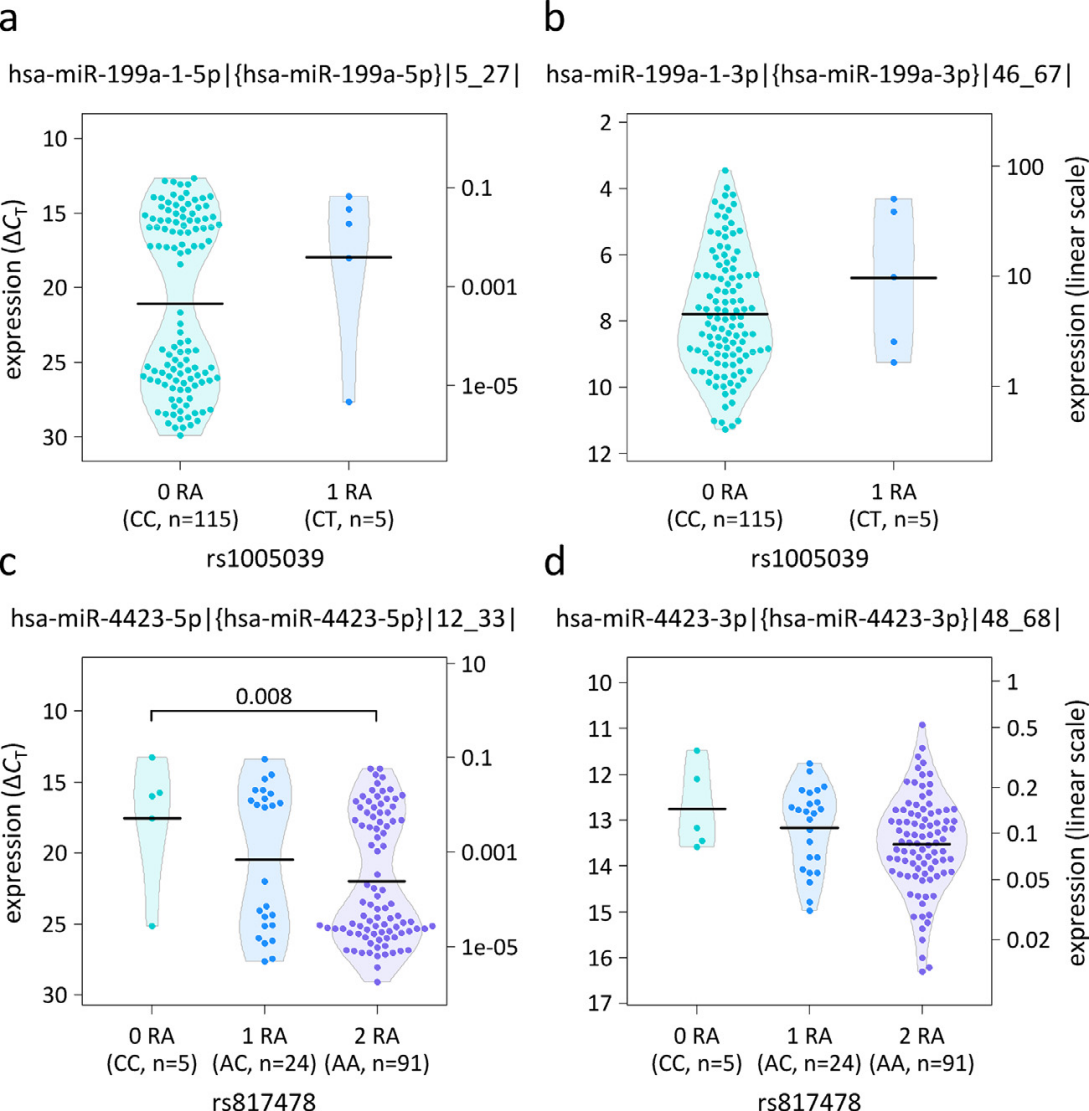
Levels of hsa-miR-199a-5p and -3p as well as hsa-miR-4423-5p and -3p per genotype. The B-cell expression levels of 4 miRBase-annotated mature miRNAs are visualised. (**a** and **b**) A non-significant tendency towards higher expression of hsa-miR-199a-5p and -3p was noted in carriers of the MS risk allele (RA) of SNP rs1005039. (**c** and **d**) The RA of SNP rs817478 was associated with a lower expression of hsa-miR-4423-5p in the *cis*-miR-eQTL analysis (Table 4). Accordingly, the respective SNPs were suspected to affect the miRNA stem-loop processing by Drosha. The number of samples from subjects carrying 0, 1 or 2 RA is indicated below each beeswarm/violin plot. The left and right y-axes refer to the raw qPCR data (Δ*C*_T_ values displayed in an inverted manner) and the converted data (2^−Δ^*^C^*^T^×1,000), respectively. The means per genotype group are shown as black lines. The Tukey test P-value reaching the significance level of α=0.05 is indicated above the bracket. eQTL=expression quantitative trait locus, miRNA=microRNA, MS=multiple sclerosis, n=number, qPCR=quantitative polymerase chain reaction, SNP=single-nucleotide polymorphism.

The *cis*-miR-eQTL analysis was conducted for hsa-mir-26a-2, hsa-mir-199a-1, hsa-mir-4304, hsa-mir-4423 and hsa-mir-4492. For these pri-miRNAs, we were able to measure corresponding mature miRNAs in at least half of the samples, on the one hand, and to detect both alleles of the MIR SNPs, on the other hand. For the genotype of SNP rs817478, we found a significant association with the levels of hsa-miR-4423-5p in B cells (Wald test P<0.05) (Table 4). The levels of the canonical miRNA and the respective 3’ isomiR were found to be significantly lower in individuals that are homozygous for the risk allele A (Tukey's test P≤0.008). A similar trend was seen for hsa-miR-4423-3p (Figure 3). We also observed higher levels of hsa-miR-199a-5p and -3p in carriers of the risk allele T of SNP rs1005039 (Figure 3). These trends did not reach statistical significance (Wald test P=0.702 and P=0.158, respectively) (Table 4), with the analysis being hampered by the low MAF. For hsa-mir-26a-2, hsa-mir-4304 and hsa-mir-4492, we did not find evidence of a genotype-dependent processing. The results were essentially the same when using the truncated data in the sensitivity analysis.

**Table 4:** Results of the analysis to detect *cis*-miR-eQTL effects in B cells.

MIR SNP	Allele frequency	RA	MicroRNA assay a	0 RA, mean ± SD (mv b)	1 RA, mean ± SD (mv b)	2 RA, mean ± SD (mv b)	P-value c
rs41292017	G: 98.9%; A: 1.1%	A	hsa-miR-26a-2-5p|{hsa-miR-26a-5p}|13_34|	2.01 ± 0.44	2.07 ± 0.64	—	0.5902
			hsa-miR-26a-2-5p|{isomiR}|13_33|	0.65 ± 0.53	0.78 ± 0.85	—	0.5059
			hsa-miR-26a-2-3p|{hsa-miR-26a-2-3p}|51_72|	13.51 ± 1.15 (10)	13.69 ± 0.83	—	0.4343
rs1005039	C: 97.8%; T: 2.2%	T	hsa-miR-199a-1-5p|{hsa-miR-199a-5p}|5_27|	21.07 ± 5.77 (59)	17.98 ± 5.64	—	0.7023
			hsa-miR-199a-1-5p|{isomiR}|5_26|	11.63 ± 2.30 (2)	10.48 ± 2.51	—	0.3546
			hsa-miR-199a-1-3p|{hsa-miR-199a-3p}|46_67|	7.78 ± 1.82	6.71 ± 2.23	—	0.1580
			hsa-miR-199a-1-3p|{isomiR}|46_66|	8.00 ± 1.46	7.07 ± 1.79	—	0.2090
rs78351440	C: 94.5%; A: 5.5%	C	hsa-miR-4304-5p|{hsa-miR-4304}|10_26|	—	10.54 ± 0.69	10.68 ± 0.88	0.7112
			hsa-miR-4304-3p|{isomiR}|35_52|	—	10.93 ± 1.09	10.98 ± 0.97	0.7410
rs817478	A: 84.6%; C: 15.4%	A	hsa-miR-4423-5p|{hsa-miR-4423-5p}|12_33|	17.56 ± 4.51 (1)	20.46 ± 5.02 (8)	21.99 ± 4.41 (46)	**0.0015**
			hsa-miR-4423-5p|{isomiR}|12_32|	18.55 ± 5.01 (2)	19.02 ± 4.15 (8)	21.13 ± 3.87 (41)	**0.0030**
			hsa-miR-4423-3p|{hsa-miR-4423-3p}|48_68|	12.75 ± 0.92	13.16 ± 0.90	13.52 ± 1.04	0.0934
			hsa-miR-4423-3p|{isomiR}|48_67|	12.68 ± 0.68	12.79 ± 0.95	13.16 ± 0.91	0.1230
rs7926599	G: 98.9%; C: 1.1%	G	hsa-miR-4492-3p|{isomiR}|58_74|	—	8.30 ± 0.43	6.59 ± 1.18	0.5171

The expression of mature miRNA molecules in B cells from the peripheral blood was measured by stem-loop qPCR assays. The *cis*-miR-eQTL analysis included only miRNAs that could be detected in at least half of the samples (n=120) and only MS-associated SNPs in miRNA-coding regions (MIR SNPs) for which both alleles were present in the data. For each MIR SNP, the allele frequencies in the study population (n=91 subjects) as well as the respective MS risk allele are given in the table. Average Δ*C*_T_ values are reported dependent on the MIR SNP genotype as specified by the number of risk alleles carried. Lower Δ*C*_T_ values correspond to higher expression levels. For 4 of the 5 MIR SNPs, there were no homozygotes for the minor allele (—). Significance values indicating *cis*-miR-eQTL effects (P<0.05) are marked in bold. — = not available, eQTL=expression quantitative trait locus, MIR/miRNA=microRNA, MS=multiple sclerosis, mv=missing values, qPCR=quantitative polymerase chain reaction, RA=risk allele, SD=standard deviation, SNP=single-nucleotide polymorphism.

a the miRNAs are specified following the nomenclature by Cloonan et al.^56^

b the number of samples in which the miRNA could not be detected within 45 qPCR cycles and for which *C*_T_ values were thus imputed.

c Wald χ^2^ test P-values for linear mixed-effects models fitted to the data in linear scale and adjusted for age, sex and group assignment.

### Verification of allele-specific primary microRNA processing efficiencies

To confirm that MS-associated genetic variants in pri-miRNA processing sites causally affect the Drosha-mediated stem-loop cleavage, a luciferase reporter system was utilised. The assay was first established with the pri-miRNA hsa-mir-16-1. The wild-type hsa-mir-16-1 was efficiently processed across the entire range of experimental conditions, as reflected by much lower relative luminescence signals in comparison to the negative control (Figure S2). In contrast, the cleavage activity was completely lost when disrupting the CNNC motif in the flanking region by introducing a C>T mutation at position 3’ (+19) (ANOVA P=6.7×10^−29^).

**Figure 4:**
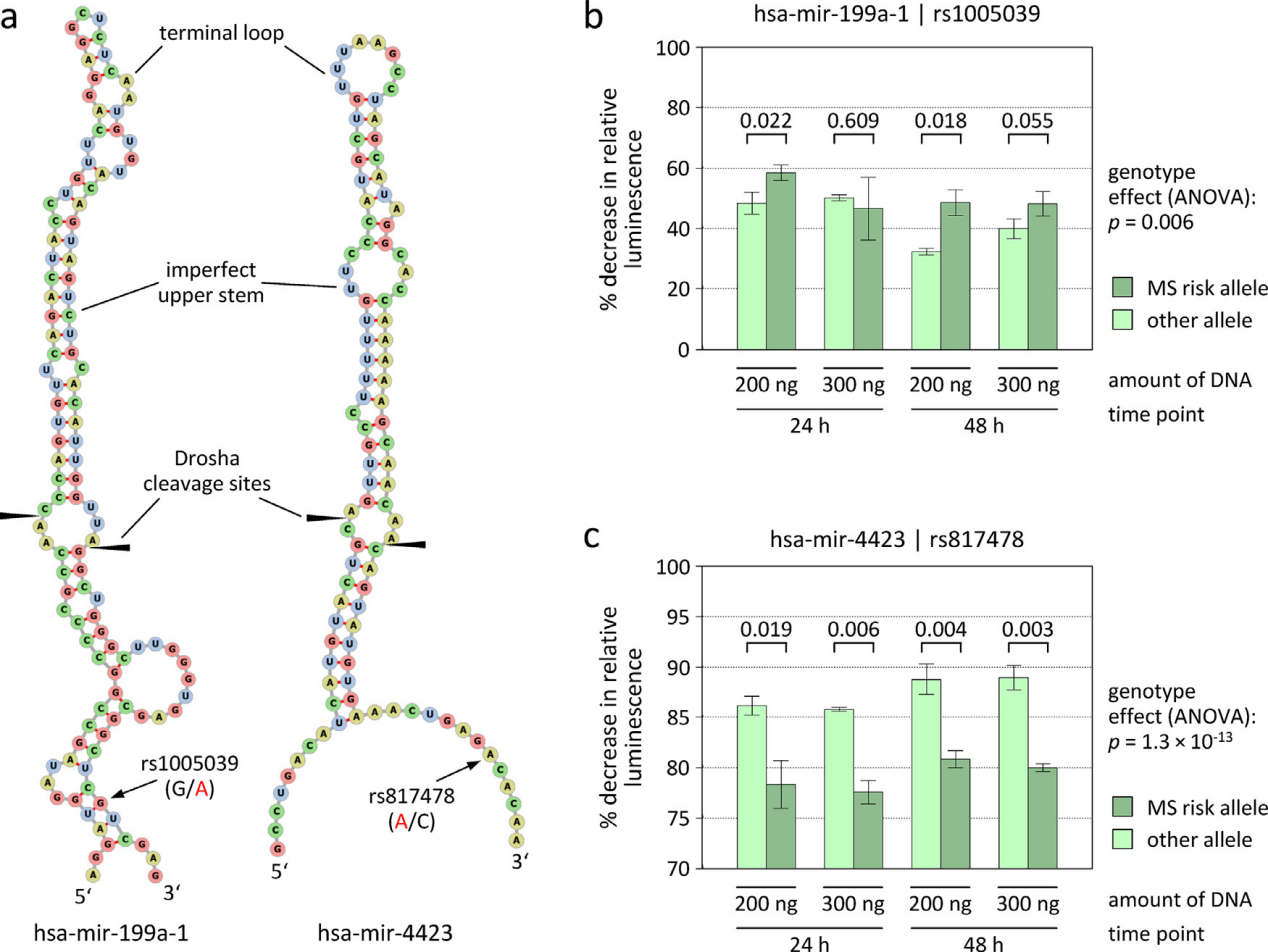
Allele-specific processing of microRNA precursor sequences in the reporter assay. **(a)** The predicted secondary structures of the miRNA stem-loops were visualised using the web-based tool forna.^58^ Both SNPs are located in a CNNC motif in the 3’ flanking region. The MS risk allele (RA) is marked in red. The Drosha cleavage sites are indicated according to the miRBase annotation.^36^ (**b** and **c**) The processing of hsa-mir-199a-1 and hsa-mir-4423 was analysed using a luciferase-based assay. For this purpose, the miRNA precursor sequences were cloned into the 3’ UTR of the GLuc gene within a reporter vector. GLuc/SEAP ratios were then measured after transient transfection of HeLa cells with plasmids carrying either the MS risk allele or the alternative allele. The decrease in relative luminescence was calculated by subtracting from 1 the quotient of the GLuc/SEAP ratio and the respective ratio that was obtained for the negative control. Higher bars thus indicate higher processing efficiencies. The bars and error bars show the means and standard deviations of 3 biological replicates each. Welch *t*-test P-values are given above the bars. The MS RA of the SNP rs1005039 conferred a higher hsa-mir-199a-1 cleavage efficiency (**b**), whereas the RA of SNP rs817478 was associated with a significantly diminished processing of hsa-mir-4423, independently of the amount of plasmid DNA used for transfection and the readout time point (**c**). ANOVA=analysis of variance (3-way additive model), GLuc=*Gaussia* luciferase, miRNA=microRNA, MS=multiple sclerosis, SEAP=secreted alkaline phosphatase, SNP=single-nucleotide polymorphism, UTR=untranslated region.

To further substantiate the results of the *cis*-miR-eQTL analysis, we then tested whether the SNPs rs1005039 and rs817478 directly implicate an altered processing of hsa-mir-199a-1 and hsa-mir-4423, respectively. The rare MS risk allele of SNP rs1005039 leads to an A in the second of two adjacent CNNC motifs in the transcript sequence (Figure 4a). In general, a stronger decrease in relative luminescence was observed when HeLa cells were transfected with the plasmid carrying the risk allele than when HeLa cells were transfected with the plasmid carrying the other allele (Figure 4b). Although this difference was not always statistically significant at individual experimental conditions in *t*-tests, the overall genotype effect was significant when analysing the whole dataset in a 3-way ANOVA (P=0.006). This points to a more efficient processing of the hsa-mir-199a-1 stem-loop in the presence of the risk allele of SNP rs1005039.

The SNP rs817478 denotes an A/C polymorphism. The C allele creates a CNNC motif downstream of hsa-mir-4423, whereas the motif is missing when the more common MS risk allele A is present. In the luciferase reporter assay, a strong decrease in relative luminescence (>75%) was generally seen after transfection of HeLa cells with GLuc-hsa-mir-4423 constructs. However, a significantly lower decrease was consistently observed when the construct carrying the risk allele was used (*t*-test P≤0.019 and ANOVA P=1.3×10^−13^). This clearly indicates that the MS risk allele of SNP rs817478 is associated with a much lower efficiency of hsa-mir-4423 processing by Drosha (Figure 4c).

We also assessed whether the SNPs rs41292017, rs78351440 and rs7926599 affect the cleavage of hsa-mir-26a-2, hsa-mir-4304 and hsa-mir-4492, respectively. However, in line with the results of the *cis*-miR-eQTL analysis (Table 4), there was no evidence of a genotype-dependent processing of these miRNA stem-loops in the reporter assays (3-way ANOVA P>0.125).

### Target genes of hsa-mir-199a-1 and hsa-mir-4423

The *cis*-miR-eQTL analyses and the luciferase reporter assays suggested that MS-associated SNPs modulate the processing of hsa-mir-199a-1 and hsa-mir-4423. To identify their target genes, we transfected HeLa cells either with the respective precursor miRNA expression plasmids, which did not carry the MS risk allele but the other allele, or with the scrambled control plasmid. The qPCR analysis revealed that the endogenous expression of the mature miRNAs in HeLa cells was low or absent. In contrast, a massive overexpression of the miRNAs was detected 24 h and 48 h after transfection with the precursor miRNA plasmids (Table S3).

The subsequent microarray analysis disclosed the expression levels of 135,750 different TCs. The transcriptome data were of high quality and showed a similar distribution across all 18 samples (Figure S3). Compared to the negative controls, 152 TCs were expressed at significantly lower levels when hsa-mir-199a-1 was overexpressed (P<0.05 and fold-change <−1.5). Of those, 60 transcripts were found to encode proteins and to contain at least one predicted binding site for the respective 5p or 3p mature miRNAs in their 3’ UTRs (Table 5). For HeLa cells that were transfected with the hsa-mir-4423 plasmid, we filtered 62 TCs with lower expression (P<0.05 and fold-change <−1.5). However, of those, *TMEM47* was the only protein-coding transcript with sequence complementarity in the 3’ UTR to the corresponding mature miRNAs. The mRNA expression fold-changes and P*-*values as well as the estimated minimum hybridisation energies are provided for all prioritised target genes in Table S4. Information on their expression in cell types of the blood and brain is given in Table S5. The transcriptome data are available in the GEO database (accession number GSE185859).

**Table 5:** Prioritised target genes of hsa-mir-199a-1 and hsa-mir-4423.

Primary microRNA	N	Prioritised target genes	Gene functions
hsa-mir-199a-1	60	*ACTN4, AMZ2, ARHGAP31, ARPC1A, BMP1, CARD19, CD9, CDK4, CIZ1, CSNK1G2, CSRNP1, DYRK1B, EEF2, EFNA1, EPSTI1, FAIM, FEN1, FXR2, HEATR3, HTRA2, IER3, IRAK2, MAP3K12, MIF, MLX, MRPS36, MTG2, NACC1, NDUFA11, NT5C, OGFOD3, OR8B3, PACS1, PEA15, PFKP, PNPLA6, PON2, PPIB, PRMT1, PROCR, RAB8A, RAD9A, RAP2B, RPS29, SIRT6, SPATA2, TAC1, TCEAL3, TMOD2, TNFRSF12A, TPRA1, TRAF1, TUBB6, UBE2D1, VIM, ZDHHC3, ZFYVE27, ZNF511, ZNF526, ZNF579*	Reactome pathways: Disease, Immune system, Infectious disease, Metabolism, Signal transduction Gene Ontology categories: Cellular protein modification process, RNA binding
hsa-mir-4423	1	*TMEM47*	UniProt: Cell junction organisation

We obtained transcriptome profiles of HeLa cells in which the respective miRNA precursor transcripts were overexpressed. Listed are the potential direct target genes identified by this screening that encode proteins and for which a hybridisation of their 3′ UTR sequences with the mature miRNA sequences could be computationally verified. The online tools Reactome^79^ and Enrichr^80^ were used to analyse the involvement of the target genes of hsa-mir-199a-1 in biological processes. See Table S4 and Table S6 for further information. miRNA=microRNA, N=number, UTR=untranslated region.

A subset of 26 of the 60 target genes that we identified for hsa-mir-199a-1 were also predicted as putative targets in the miRWalk database,^77^ and *NACC1* and *TMOD2* are already listed as experimentally verified targets in the miRTarBase database.^78^ The target genes of hsa-mir-199a-1 were found to participate in diverse biological functions, including immune system signalling, metabolism and RNA binding. *TMEM47*, the prioritised target gene of hsa-mir-4423, is a transmembrane protein that regulates the organisation of cellular junctions. Interactions of hsa-miR-4423-5p and -3p with the 3’ UTR of *TMEM47* were also predicted by miRWalk^77^ (Tables 5 and S6).

## Discussion

Until now, about 200 genetic loci have been linked to the inherited risk of developing MS,^26^ but our functional understanding of the underlying molecular mechanisms is still scarce. Here, we hypothesised that pri-miRNA processing sites contain causal variants for this disease. We combined expression analyses of B cells, experiments in cell culture and *in silico* evaluations to shed light on the maturation and function of largely underexplored and primate-specific miRNAs. To our knowledge, at least the prioritised miRNAs with 4-digit numbers have never been explicitly measured in B cells from MS patients before. We found that the Drosha-mediated cleavage of hsa-mir-199a-1 and, in particular, hsa-mir-4423 is influenced by proximal MS risk variants. We have also determined potential direct target genes of these miRNAs.

We first examined the association between the genotype of MS-associated SNPs and the levels of mature miRNAs and isomiRs in B cells. This was done in a representative study population, with the exception that we did not include patients with SPMS. Otherwise, the patient cohort reflected typical characteristics of MS patients in terms of age, sex and disease status, resembling those of patients included in European MS registries.^88^ With flow cytometry, we confirmed a strong enrichment of B cells in the samples, while the percentages of the 8 B-cell subpopulations were within the expected ranges: The distribution of B-cell subsets was quite similar when comparing healthy subjects with the group of RRMS patients before IRT. Patients receiving natalizumab infusions had a lower proportion of naive B cells but a higher proportion of memory B cells in the blood, as previously described.^89^^,^^90^ In contrast, patients who were treated with oral cladribine or intravenous alemtuzumab showed a relative increase in the frequency of transitional and naive B cells and a decrease in the frequency of memory B cells. This is in line with earlier studies that have shown that B-cell repopulation following infusion of alemtuzumab or (subcutaneous) administration of cladribine results in such shifts in B-cell subpopulations.^15^^,^^16^^,^^91^^,^^92^ However, the effects of these IRTs for MS on miRNA expression have not been investigated so far.

Compared to the healthy controls, we observed a significant differential expression of hsa-miR-26a-5p and -3p in RRMS patients treated with natalizumab and alemtuzumab, respectively. In previous studies, altered levels of hsa-miR-26a-5p were detected in serum,^93^^,^^94^ whole blood,^95^ PBMC^96^, ^97^, ^98^ and thrombocytes^99^ of patients with MS. However, inconsistent results have been described, most likely due to different study designs. For instance, in response to interferon-β therapy, higher levels of this miRNA were reported in thrombocytes,^99^ but lower levels were reported in serum exosomes.^94^ Our study differs from the existent studies in that we explicitly analysed B cells, that we also measured hsa-miR-26a-3p levels and that we included patients who received IRTs. The pri-miRNA hsa-mir-26a-2 is encoded on chromosome 12 and belongs to the mir-26 family, which is conserved in vertebrates.^36^ Of note, hsa-miR-26a-5p is also produced from the hsa-mir-26a-1 locus on chromosome 3, which weakened the validity of the *cis*-miR-eQTL analysis. There is accumulating evidence that miR-26a miRNAs play pivotal roles in cellular differentiation, cell growth and apoptosis and that they are involved in the development of various human diseases.^100^ In experimental autoimmune encephalomyelitis (EAE), an animal model of MS, silencing of miR-26a-5p was found to result in increased expression of T_h_17-related cytokines and increased disease severity, while overexpression of the precursor miRNA was found to result in reduced expression of T_h_17-related cytokines and a milder form of EAE.^95^ A direct target of miR-26a-5p is IL6 mRNA.^95^

We measured higher levels of hsa-miR-199a-5p and -3p in B cells from alemtuzumab-treated patients than in those from healthy controls. The literature on the expression of these miRNAs in MS patients is heterogeneous, with previous studies performed with sera,^101^ PBMC,^102^ CD4^+^ T cells,^103^ cerebrospinal fluid samples^104^ and brain lesion tissues.^105^ Remarkably, the levels of hsa-miR-199a-5p were described to be ∼3 times higher in CD4^+^ T cells of RRMS patients in remission compared to healthy subjects^103^ and in MS lesions compared to normal brain white matter.^105^ The mir-199 family is evolutionarily highly conserved, and it should be noted that the -5p and -3p mature miRNA molecules are produced from two loci (hsa-mir-199a-1 on chromosome 19 and hsa-mir-199a-2 on chromosome 1).^36^ Functional studies have demonstrated that especially the -3p mature form may act as either a tumour suppressor or an oncogene, depending on the type of cancer.^106^ The miR-199a miRNAs were shown to regulate tumour cell proliferation, invasion, apoptosis and glucose metabolism, to protect against hypoxia-induced damage in cardiomyocytes, to modulate stem cell differentiation and fetal development and to affect adipocyte differentiation and fatty acid composition.^106^, ^107^, ^108^

In our study, only 5 B-cell samples were heterozygous for the MS-associated allele T of SNP rs1005039 that is located in one of two CNNC motifs flanking hsa-mir-199a-1. The low MAF possibly accounts for the fact that the suspected *cis*-miR-eQTL effect could not be detected with statistical significance. However, the results of the luciferase reporter assays indicated a higher pri-miRNA processing efficiency in HeLa cells when the MS risk allele was present. Likewise, Roden *et al.* have demonstrated that an exchange of the NN bases within CNNC can have a mild effect on the stem-loop processing.^47^ Moreover, a previous study reported that the C allele of SNP rs3760781, which has a distance of ∼19 kb to the pri-miRNA locus, is associated with significantly higher levels of hsa-miR-199a-3p and -5p in plasma.^109^ The worldwide frequency of the haplotype TCC of the MIR SNP (rs1005039), the SNP from ^109^ (rs3760781) and the MS lead SNP (rs12609500) is 1.3% according to the 1000 Genomes data.^51^^,^^52^ In such a context, a small sample size, as in our study, implies high uncertainty in the inferences that can be drawn.^110^ Hence, to further substantiate the presumed causal relationship between genetic risk, miRNA expression and MS, a larger study population and/or a family-based approach is needed. A number of target genes of miR-199a miRNAs have been already experimentally determined in previous studies, as recorded in the miRTarBase database.^78^ In the present study, 60 protein-coding target genes were prioritised for hsa-mir-199a-1, some of which are novel. For instance, the immune-related genes *IRAK2, MIF* and *VIM* were not contained as known or predicted targets in the databases miRTarBase^78^ and miRWalk,^77^ respectively. These and other target gene candidates that were identified by our transcriptome screening await further confirmation in future studies.

The MS risk allele A of SNP rs817478 was associated with significantly lower levels of hsa-miR-4423-5p in B cells in our *cis*-miR-eQTL analysis. The same trend was seen for hsa-miR-4423-3p. In the reporter assay experiment, an allele-dependent cleavage rate of hsa-mir-4423 was clearly confirmed across all experimental conditions. These data thus suggest a causal role of miR-4423 miRNAs in the genetic predisposition to MS. The association of the rs817478 genotype with hsa-miR-4423-5p expression was previously identified as one of 57 *cis*-miR-eQTLs in the European subset of the Geuvadis RNA-sequencing data on EBV-transformed lymphoblastoid cell lines.^33^ The eQTL is also contained in the ncRNA-eQTL database, which is solely based on cancer-related studies.^111^ The genetic effect is explained by the fact that there is a 3′ flanking CNNC motif in the presence of the minor allele C but not in the presence of the MS risk allele A. This sequence feature is well known to enhance the Drosha-mediated processing and is found downstream of one third of all human conserved miRNA stem-loops.^44^^,^^47^ The CNNC motif is bound by SRSF3, which is a major auxiliary factor for pri-miRNA processing,^112^ and both hsa-mir-199a-1 and hsa-mir-4423 were found to be productively processed by Drosha in the presence of SRSF3.^113^ The pri-miRNA hsa-mir-4423 has recently evolved: It is currently only annotated in the genomes of humans and chimpanzees.^36^ The mature miRNA from the 3′ arm has been first identified in malignant B cells.^114^ Perdomo et al. have shown that hsa-mir-4423 is a regulator of airway epithelial cell differentiation and a repressor of lung carcinogenesis.^115^ Otherwise, its function is still largely unclear. The miRTarBase database currently contains no miRNA-target interaction with strong evidence (*i.e.*, with verification by qPCR, western blot or reporter assay) for hsa-mir-4423.^78^ Our analysis revealed only *TMEM47* as potential direct target gene of this miRNA. This gene was also found to be significantly lower expressed in SW900 cells overexpressing hsa-mir-4423 in the data from Perdomo et al. (GEO accession GSE48796).^115^ TMEM47 is an adherens junction protein involved in regulating epithelial cell junction organisation.^116^ It is also upregulated during astrocyte maturation,^117^ but it remains to be clarified to which degree it plays a role in MS. Further investigations on the cell type-specific functions of this gene are warranted.

Several limitations of this study should be considered in the data interpretation. First, the patients in our study were heterogeneous with respect to the treatment they received. We accounted for this by including the group assignment in the *cis*-miR-eQTL analysis, while also adjusting for age and sex, but the possibility of unmeasured confounding cannot be ruled out. It also remains unclear whether other IRTs, such as ocrelizumab, affect the expression of the miRNAs. Second, the measurement of mature miRNA levels was conducted in B cells, and we restricted our analysis to miRNAs that could be detected in at least half of the samples. However, miRNAs from hsa-mir-3661 and hsa-mir-4252 could not be assayed properly, and other miRNAs were barely detectable, including hsa-miR-548ac-3p, for which we have previously shown a *cis*-miR-eQTL effect and which is preferentially expressed in CD8^+^ T cells and natural killer cells.^48^^,^^118^ We therefore presumably missed eQTLs for miRNAs that are expressed predominantly in other cells as well as eQTLs that are cell type-specific, a phenomenon that has been studied more thoroughly for protein-coding genes.^119^^,^^120^ Deeper insights on the impact of the genetic risk variants might be achieved by examining other peripheral immune cells, CNS-resident cells and/or specific B-cell subsets, e.g., memory B cells. Third, our study was limited to selected SNPs and miRNAs. We did not include rare variants with MAF<1% because the sample size would have been insufficient to study *cis*-miR-eQTL associations. We also excluded miRNAs that are produced independently of Drosha (e.g., mirtrons^39^). Moreover, it is difficult to define possible isomiRs and to measure their expression accurately. Here, we employed stem-loop RT-qPCR assays. These assays allow to discriminate between closely related mature miRNAs,^121^ but different 3’ isomiR species may be cross-detected.^122^ The exclusive measurement of miRNA isoforms that differ by only 1 nt at their termini is possible by dumbbell PCR with TaqMan probes,^123^ but there are currently no commercial assays for this. Fourth, the efficiency of pri-miRNA processing was evaluated using reporter assays in which transiently expressed luciferase mRNA is cleaved by endogenous Microprocessor in HeLa cells.^69^^,^^70^ As an alternative, recombinant Drosha and DGCR8 proteins can be used in kinetic assays to more precisely determine the allele-specific rates at which the cleavage occurs.^124^ It is important to note, however, that such assays do not reflect all regulatory events taking place *in vivo*. In fact, various mechanisms coordinate the biogenesis and turnover of mature miRNAs (e.g., Dicer activity and miRNA decay^39^). Further research is needed to elucidate how the interplay of genetic factors with environmental and lifestyle factors (e.g., EBV infection and vitamin D deficiency) may alter molecular networks related to miRNA maturation and function.^125^^,^^126^ Fifth, a transcriptome-wide analysis was conducted to identify the most likely direct target genes of hsa-mir-199a-1 and hsa-mir-4423. However, we might have missed actual target genes, e.g., those that are not endogenously expressed in HeLa cells and those that are inhibited by translational repression rather than mRNA destabilisation. In future studies, improved experimental strategies for miRNA target identification may be applied to verify effects at the protein level and to better resolve the functional differences between miRNA family members and isoforms.^127^

In conclusion, we here focused on miRNAs that are encoded in MS-associated genetic regions. We observed a significant differential expression of mature miRNAs and isomiRs from hsa-mir-26a-2 and hsa-mir-199a-1 in B cells when comparing subgroups of RRMS patients and healthy controls. Based on *cis*-miR-eQTL analyses and reporter assays, we found that the MS risk allele T of SNP rs1005039 is related to a somewhat more efficient Drosha-dependent processing of hsa-mir-199a-1, whereas the MS risk allele A of SNP rs817478 confers a much lower hsa-mir-4423 processing efficiency. These findings complement the existing body of literature on the dysregulation of miRNAs in MS and on sequence determinants of pri-miRNA processing. Our and other data indicate that diverse biological processes are regulated by hsa-mir-199a-1, while the role of hsa-mir-4423 remains poorly defined. For a deeper understanding of the pathomechanisms of MS, further functional studies are needed to identify the disease-causing genetic variants and to ascertain their effects on transcriptional activity and RNA processing.

## Contributors

MH, BF and UKZ conceptualised the study. MH, NB, EP and UKZ secured the research funding. MS, AW, SM and AD coordinated the blood sampling and collected clinical information. NB, EP and BF processed the blood samples. NB executed the majority of the experiments. DK performed the microarray analysis. PL provided valuable suggestions for the reporter assays. NB and MH analysed and interpreted the data and prepared the figures and tables. UKZ supervised the research and provided important intellectual content. MH drafted the original manuscript, and all authors reviewed, edited and prepared the final version. MH and NB had full access to all the data and take responsibility for the integrity and accuracy of the data analysis. EP, BF and DK have verified the underlying data. All authors have read and approved the final version of the manuscript.

### Data sharing statement

The data supporting the findings of this study are available in the article and/or supplementary materials. The transcriptome data from the target gene analysis are publicly available from the GEO database (https://www.ncbi.nlm.nih.gov/geo/query/acc.cgi?acc=GSE185859). Readers are welcome to contact the corresponding author by e-mail to request access to the raw de-identified data used in this work.

## Declaration of interests

MH received speaking fees and travel funds from Bayer HealthCare, Biogen, Merck, Novartis and Teva. AW received speaking fees and travel funds from Biogen, GlaxoSmithKline, Merck Serono, Novartis and Sanofi Genzyme. NB received travel funds from Novartis. UKZ received research support as well as speaking fees and travel funds from Alexion, Almirall, Bayer HealthCare, Biogen, Bristol Myers Squibb, Janssen, Merck Serono, Novartis, Roche, Sanofi Genzyme, Teva as well as EU, BMBF, BMWi and DFG. BF, EP, MS, SM, AD, DK and PL declare that they have no competing interests.

## Appendix Supplementary materials

Image, application 1

Image, application 2

Image, image 3

Image, image 4

Image, image 5
