# Supplementary material for: Implication of genetic variants in primary microRNA processing sites in the risk of multiple sclerosis
Source: eBioMedicine. 2022 May 10;80:104052. doi: 10.1016/j.ebiom.2022.104052 (PMC9111935; doi:10.1016/j.ebiom.2022.104052)
Supplement: Supplementary file 1 [file mmc1.docx]

# Supplementary material for

**Analysis of genetic variants in primary microRNA processing sites
as potential contributors to the risk of developing multiple sclerosis**

Michael Hecker^1,*^, Brit Fitzner^1^, Elena Putscher^1^, Margit Schwartz^1^, Alexander Winkelmann^1^, Stefanie Meister^1^, Ales Dudesek^1^, Dirk Koczan^2^, Peter Lorenz^2^, Nina Boxberger^1,$^,
Uwe Klaus Zettl^1,$^

^1^ Rostock University Medical Center, Department of Neurology, Division of Neuroimmunology,
Gehlsheimer Str. 20, 18147 Rostock, Germany

^2^ Rostock University Medical Center, Institute of Immunology, Schillingallee 70,
18057 Rostock, Germany

^*^ corresponding author: Michael Hecker, e-mail: michael.hecker@rocketmail.com,
phone: +49 381 494-5890, fax: +49 381 494-5882,
ORCID ID: 0000-0001-7015-3094

^$^ these authors contributed equally to this work

**Figure S1** : Gating strategy for B-cell phenotyping.

Shown are exemplary data obtained by flow cytometry of thawed cryopreserved cells of a healthy subject. The plots visualise all events that resulted from the gating of live single CD19^+^ cells. B-cell subpopulations were identified by the markers CD21, CD23, CD24, CD27, CD38 and IgD, basically following the gating strategy described by Morbach *et al.* (Clin Exp Immunol, 2010). (**a**) CD27 was plotted against IgD to identify CD27^+^IgD^−^ switched memory B cells, CD27^+^IgD^+^ non-switched memory B cells, CD27^−^IgD^−^ memory B cells and CD27^−^IgD^+^ naive B cells. (**b**) Transitional B cells were defined as CD24^++^CD38^++^ cells and plasmablasts as CD24^−^CD38^++^ cells. Additionally, CD21^−/low^CD38^−/low^ B cells (**c**) and CD23^+^ B cells (**d**) were gated. Numerical values in the gates indicate percentages relative to all live single CD19^+^ cells. BC = B cells, FSC-A = forward scatter area.

**Figure S2** : Establishment of a reporter assay to assess primary microRNA processing efficiencies.

(**a**) Secondary structure of hsa-mir-16-1 as predicted using forna (Kerpedjiev *et al.*, Bioinformatics, 2015). The mutation altered the first C in the CNNC motif. The Drosha cleavage sites are indicated according to the miRBase annotation (Kozomara *et al.*, Nucleic Acids Res, 2019). (**b**) The luciferase-based assay was adapted from Allegra *et al*. (Biochem Biophys Res Commun, 2011) and tested with the well-studied primary miRNA hsa-mir-16-1 (Auyeung *et al*., Cell, 2013). Plasmids were constructed to express the miRNA stem-loop sequence together with GLuc as a combined transcript. A C>T mutation at position 3' (+19) was introduced to disrupt the CNNC motif, which is a key determinant of primary miRNA recognition and processing (Kim *et al*., Mol Cell, 2021). The original luciferase reporter vector without precursor miRNA sequence served as negative control. Different amounts of DNA were used for the transfection of HeLa cells, and GLuc and SEAP activities were measured at 3 different time points post-transfection. The means and standard deviations of the GLuc/SEAP ratios from 3 biological replicates per experimental condition are visualised. Welch *t*-test *p*-values are given above the bars. The relative luminescence was consistently and significantly lower for the wild-type hsa-mir-16-1, indicating a high cleavage activity. In contrast, the mutated form led to similarly high GLuc/SEAP ratios as the negative control, demonstrating that the stem-loop processing was completely abolished. ANOVA = analysis of variance (3-way additive model), GLuc = *Gaussia* luciferase, miRNA = microRNA, SEAP = secreted alkaline phosphatase.

**Figure S3** : Quality control of the transcriptome data.

HeLa cells were transiently transfected with two precursor microRNA (miRNA) expression vectors or the control plasmid with scrambled sequence. Eighteen high-density Clariom D arrays were then used to identify target genes of hsa-mir-199a-1 and hsa-mir-4423 at 24 h and 48 h post-transfection. (**a**) Signal boxplot of the microarray data before normalisation. The boxes define the upper and lower quartiles of all signal intensities in log2 scale. The medians are shown as horizontal lines. Upper and lower whiskers indicate the 90% and the 10% quantiles, respectively. Overall, the raw data distribution was similar across the samples. (**b**) Evaluation of hybridisation efficiencies. Staggered amounts of hybridisation controls were spiked into the hybridisation cocktail. These are biotinylated RNA molecules derived from biotin synthesis pathway genes of *Escherichia coli* (BioB, BioC and BioD) and the Cre recombinase of bacteriophage P1. The line graph shows the expected relative increase in signal from BioB to Cre. (**c** and **d**) Verification of overexpression of miRNA precursors. Two Clariom D probe sets (also referred to as transcript clusters, TC) comprise oligonucleotide probes that match to the sequence of the stem-loop formed by the primary transcript of hsa-mir-199a-1 (**c**) and hsa-mir-4423 (**d**), respectively. Shown are the Tukey biweight averages of the measured expression levels after transfection with the 3 different plasmids.
