# Supplementary material for: Implication of genetic variants in primary microRNA processing sites in the risk of multiple sclerosis
Source: eBioMedicine. 2022 May 10;80:104052. doi: 10.1016/j.ebiom.2022.104052 (PMC9111935; doi:10.1016/j.ebiom.2022.104052)
Supplement: Supplementary file 2 [file mmc2.docx]

**Table S1.** Comparison of B-cell subpopulation frequencies between the study groups.

Flow cytometry was used to determine the percentages of 8 B-cell subpopulations of enriched CD19^+^ B cells from 120 blood samples that were obtained from healthy individuals as well as MS patients with different disease courses and different therapies. For all subpopulations, there was a significant difference in the percentages when comparing the groups (P<0.05). IRT=immune reconstitution therapy, MS=multiple sclerosis, n=number, PPMS=primary progressive multiple sclerosis, RRMS=relapsing-remitting multiple sclerosis, SD=standard deviation.

^a^=*F*-test P-values for linear models adjusted for age and sex

**Table S2.** Genotype distribution of the investigated SNPs for the MS patients and controls.

Genotyping of 12 variants within or near miRNA-coding sequences (MIR SNPs) and 2 tag SNPs for HLA alleles was performed for all 91 subjects. Allele frequencies for the entire study population are reported. The MS risk allele is always specified with respect to the forward strand of the reference genome. The distributions of the genotypes of cases and controls are shown: 2 RA=homozygous for the risk allele, 1 RA=heterozygous, 0 R=homozygous for the other allele. The association between risk alleles and MS in our cohort was calculated. Significant associations (P<0.05) are marked in bold. HLA=human leukocyte antigen, MIR/miRNA=microRNA, MS=multiple sclerosis, RA=risk allele, SNP=single-nucleotide polymorphism.

^a^=this SNP is located on the sex-determining X chromosome; males carrying the RA were counted like homozygous females for the sake of simplicity

^b^=due to assay design restrictions, rs116807677 was used instead of rs77896647; both SNPs are in perfect linkage disequilibrium in the 1000 Genomes panel (Machiela *et al*., Bioinformatics, 2015)

^c^=the HLA allele and the C allele of the tagging SNP rs2844821 are considered protective in MS

^d^=one-tailed Fisher's exact test *p*-values

**Table S3**. Expression of mature microRNA molecules in transfected HeLa cells.

HeLa cells were transiently transfected with precursor miRNA expression vectors or with a negative control vector with scrambled sequence. The HeLa cells were lysed after 24 h or 48 h, and the isolated RNA samples were analysed using stem-loop RT primers and TaqMan qPCR assays to measure the levels of 4 mature miRNAs as annotated in miRBase (Kozomara *et al.*, Nucleic Acids Res, 2019). Average Δ*C*_T_ values are reported as a measure of the miRNA expression relative to the reference miRNA hsa-miR-191-5p. Low Δ*C*_T_ values indicate high miRNA levels. Negative Δ*C*_T_ values resulted when the expression level of the miRNA of interest was even higher than the expression level of the reference miRNA. If the miRNAs could not be detected in all samples within 45 qPCR cycles, the number of samples in which the molecule could not be detected is indicated in parentheses. Such missing values were only obtained for HeLa cells that were transfected with the scrambled control vector. —=not measured, h=hours, miRNA=microRNA, mv=missing values, NA=not available, qPCR=quantitative polymerase chain reaction, RT=reverse transcription, SD=standard deviation.

**Table S4**. Details on the screening for potential target genes of hsa-mir-199a-1 and hsa-mir-4423.

Clariom D arrays were used to measure the transcriptome of HeLa cells, which were transiently transfected with either a plasmid for overexpression of the precursor miRNAs or a negative control plasmid. The Transcriptome Analysis Console (TAC) version 4.0.2 was used to filter transcripts whose level is significantly lower in cells overexpressing the respective miRNA compared with the negative controls (P<0.05 and fold-change <−1.5 after 24 h or 48 h). This table provides the official gene symbols, probe set identifiers, robust means (Tukey’s biweight of log2 signals) for each group of samples (at least duplicates) as well as the results of the differential gene expression analysis. The fold-changes are given for the data on linear scale, and the negative sign indicates higher levels in the negative control samples. The RNAhybrid webserver (Krüger *et al*., Nucleic Acids Res, 2006) was used to calculate the minimum free energies (mfe) for the binding of the mature miRNAs (from the 5’ or 3’ arm of the precursor miRNAs and including potential isoforms) at the 3' UTRs of the candidate target genes. It is also reported whether there was evidence of an interaction (**✔**) in the databases miRWalk version 3.0 (Sticht *et al*., PLoS One, 2018) or miRTarBase release 8.0 (Huang *et al*., Nucleic Acids Res, 2020). h=hours, HGNC=HUGO Gene Nomenclature Committee, ID=identifier, miRNA=microRNA, UTR=untranslated region.

^a^=P-values computed using the limma method with eBayes correction (Ritchie *et al*., Nucleic Acids Res, 2015)

**Table S5**. Cell type-specific expression of the potential target genes.

Check marks (**✔**) specify whether the genes are expressed in different cell types of the blood and brain. The data on cell populations of the peripheral blood were retrieved from the GEO database (accession number GSE24759) (Novershtern *et al*., Cell, 2011). The brain cell type-specific gene expression data were downloaded from http://celltypes.org/brain/ (McKenzie *et al*., Sci Rep, 2018). A transcript level <100 was regarded as not expressed (—). Some of the genes were not available in the respective datasets (NA). DC=dendritic cell, HGNC=HUGO Gene Nomenclature Committee, ID=identifier, NK cell=natural killer cell, NKT cell=natural killer T cell, Oligodendro.= oligodendrocyte, OPC=oligodendrocyte precursor cell.

**Table S6.** Overlap of hsa-mir-199a-1 target genes with functional categories.

Listed are Reactome pathways (Jassal *et al*., Nucleic Acids Res, 2020) and Gene Ontology gene sets (The Gene Ontology Consortium, Nucleic Acids Res, 2019) that contain several of the potential direct target genes of hsa-mir-199a-1. N=number.
