# Supplementary figures and images for: Implication of genetic variants in primary microRNA processing sites in the risk of multiple sclerosis
Source: eBioMedicine. 2022 May 10;80:104052. doi: 10.1016/j.ebiom.2022.104052 (PMC9111935; doi:10.1016/j.ebiom.2022.104052)

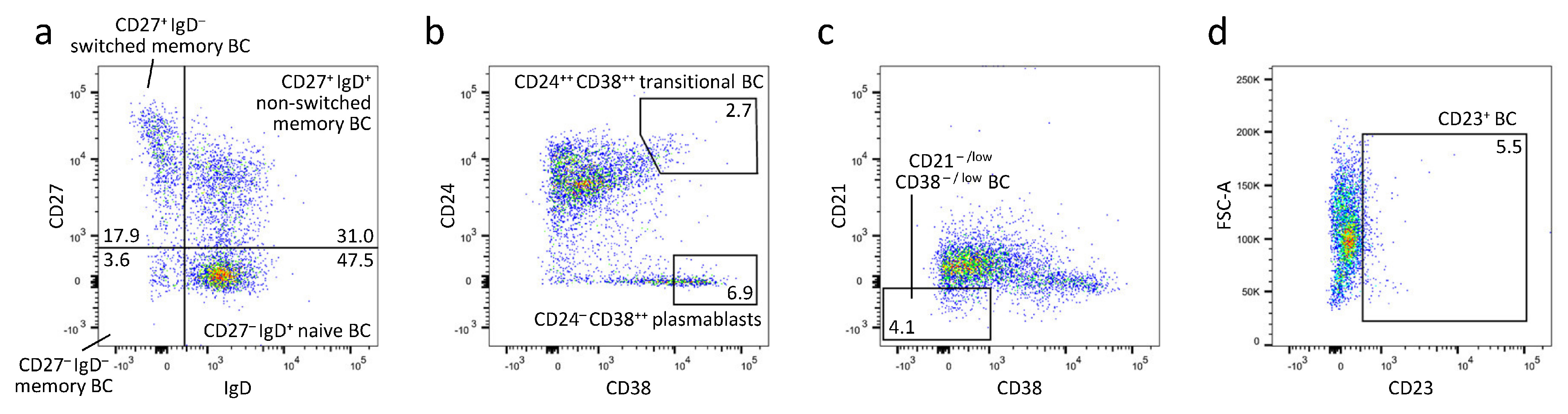

Supplement: Supplementary file 3 [file mmc3.jpg]

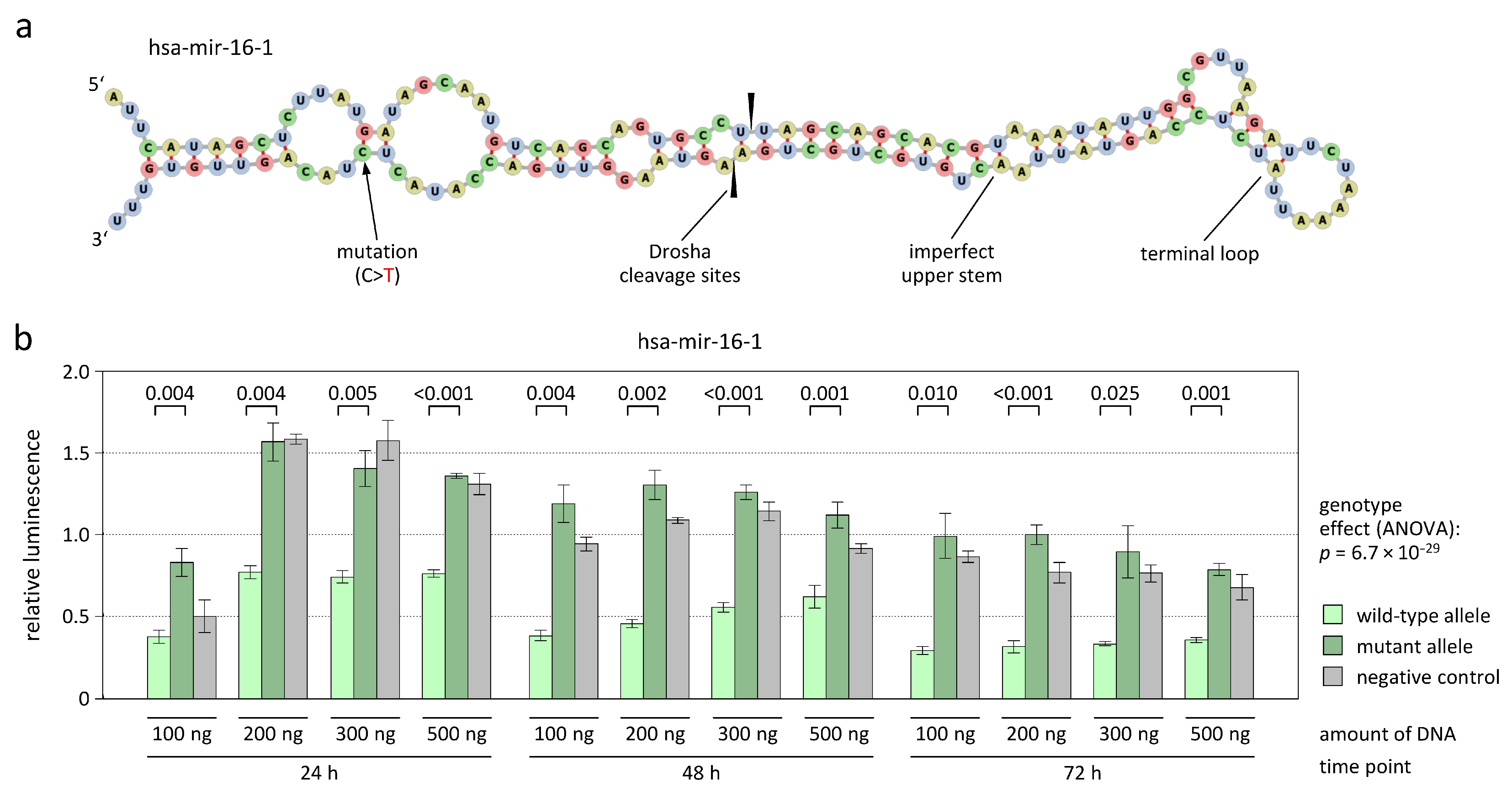

Supplement: Supplementary file 4 [file mmc4.jpg]

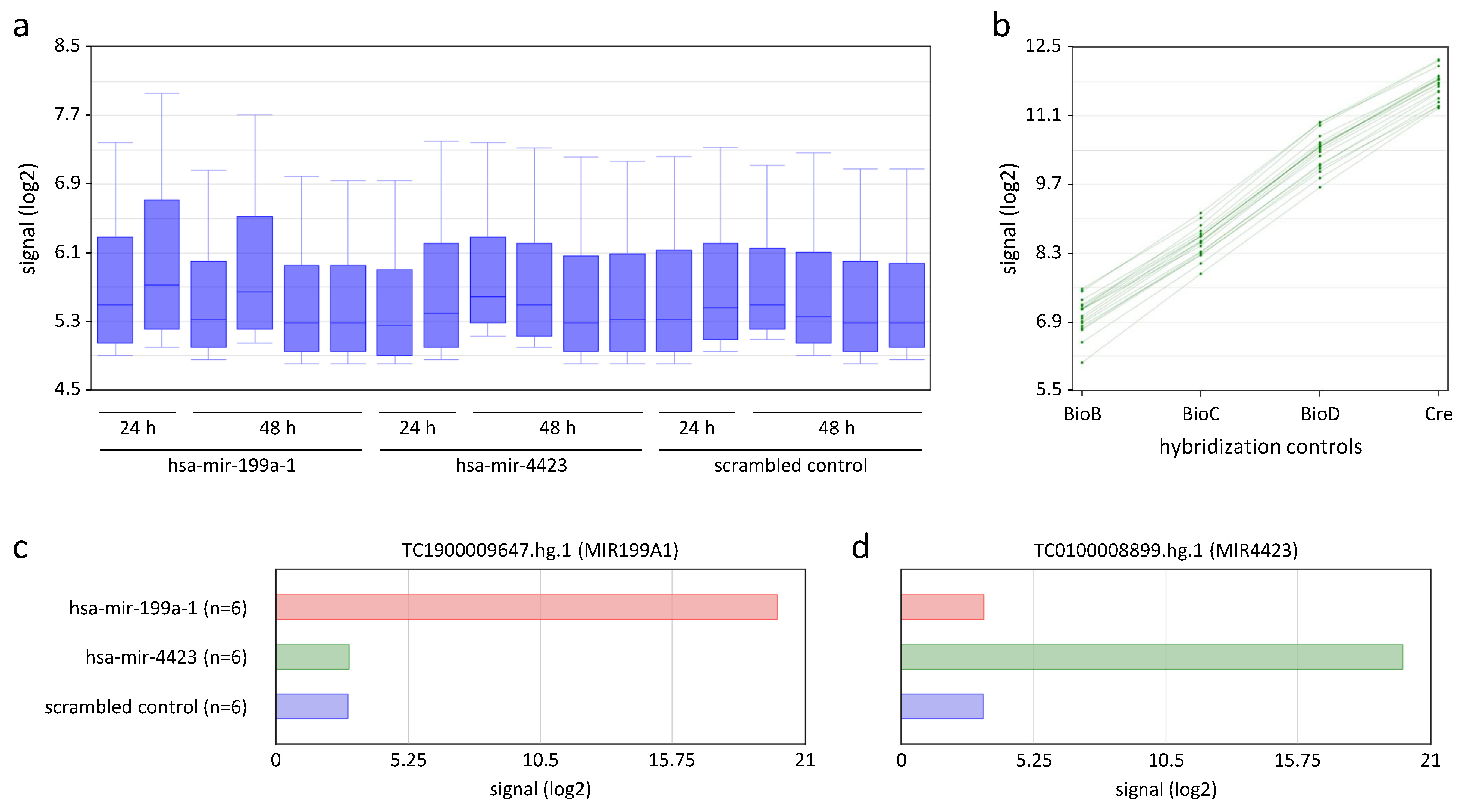

Supplement: Supplementary file 5 [file mmc5.jpg]
